# Exploring viral diversity in diarrheic porcine feces: a metagenomic analysis from an Indian swine farm

**DOI:** 10.3389/fcimb.2025.1653342

**Published:** 2025-09-12

**Authors:** Sushila Maan, Kanisht Batra, Jeyaprakash Rajendhran, Raison Joseph, Vikash K. Singh, Deepika Chaudhary, Swati Sindhu, Vijay Kadian, Aman Kumar, Narender Singh Maan, Sunil Mor

**Affiliations:** ^1^ Lala Lajpat Rai University of Veterinary and Animal Sciences (LUVAS), Haryana, India; ^2^ School of Biological Sciences, Madurai Kamaraj University, Madurai, TN, India; ^3^ Veterinary Diagnostic Lab, University of Minnesota, St. Paul, MN, United States; ^4^ Animal Disease Research & Diagnostic Laboratory, South Dakota State University, Brookings, SD, United States

**Keywords:** porcine faecal virome, metagenomics, porcine mamastrovirus, porcine circovirus, porcine parvovirus, porcine picobirnavirus, porcine posavirus, porcine picornavirus

## Abstract

**Background:**

Pig husbandry is a vital sector in India, providing nutritional security and employment for marginalized communities. Pigs are advantageous due to high reproduction rates and fecundity, shorter generation intervals, and efficient feed conversion, requiring minimal housing. However, the swine industry encounters significant disease challenges, particularly viral gastroenteritis, which poses serious public health risks, especially in developing countries. Pigs serve as natural reservoirs and amplifiers for numerous viruses with zoonotic potential, making disease surveillance essential.

**Materials:**

In this study, we conducted a metagenomic analysis of 15 fecal samples from diarrheic pigs on a farm in India, marking the first exploration of the fecal virome diversity in this region. Our next-generation sequencing approach has enabled the unbiased detection of multiple viral agents in the porcine fecal samples, detecting both known and novel viral agents without prior target knowledge.

**Results:**

The key and novel viruses obtained in our study were porcine circovirus, porcine parvovirus 7, porcine mamastrovirus 3, porcine sapelovirus A, and porcine enterovirus G. This work resulted in the generation of full genomes for multiple porcine viruses, including *Circovirus, Enterovirus, Sapelovirus*, and *Mamastrovirus*, along with partial genomes of *Parvovirus, Picobirnavirus, Porcine stool-associated RNA virus* (Porcine Posavirus), *Kobuvirus*, and *Rotavirus*, all subjected to phylogenetic analysis.

**Conclusion:**

Our survey indicates frequent co-infections with diverse viruses, creating conducive environments for viral recombination and reassortment. Continuous surveillance of viral pathogens in animal populations is essential for understanding the dynamics of both known and novel viruses and for detecting emerging pathogens, along with their zoonotic and pathogenic potential.

## 1 Introduction

Pig farming plays a vital role in supporting the rural economy and ensuring food security in rural and semi-urban communities across India. However, challenges such as a lack of knowledge, limited resources, and inadequate biosecurity measures, all of which significantly increase the risk of viral disease outbreaks, threatening both pig farming and public safety.

Over the past three decades, numerous viruses have emerged or re-emerged within the global porcine industry. While some of these viruses lead to severe clinical diseases in pigs, the economic implications of others on pig health remain uncertain. Alarmingly, certain emerging porcine viruses exhibit zoonotic potential, meaning that when these pathogens cross species barriers, they can cause devastating infections with high morbidity and mortality rates. Recent years have witnessed several outbreaks of infectious diseases in humans linked to initial zoonotic transmissions, underscoring the importance of epidemiological surveillance of animal pathogens with zoonotic potential. Emerging viruses often arise due to shifts in the interactions among the agent, host, and environment.

Porcine diarrhea significantly impacts the swine industry, frequently resulting in cases without a clearly identified viral or bacterial cause, which are often overlooked until an outbreak occurs. Notable diseases such as those caused by the porcine reproductive and respiratory syndrome virus (PRRSv), porcine epidemic diarrhea virus (PEDv), porcine circovirus type 2 (PCV-2), and the influenza H1N1 virus have led to substantial financial losses. In contrast, emerging viruses like porcine enteroviruses (PEV), porcine toroviruses (PToV), porcine sapelovirus (PSV), porcine bocavirus (PBoV), porcine kobuvirus (PKoBV), and porcine Torque teno sus virus (TTSuV) often remain subclinical in swine herds. Furthermore, newly identified viruses such as the Seneca virus, atypical porcine pestivirus (APPV), PCV-3, and influenza D present fresh challenges for researchers and veterinarians. The potential for severe disease outbreaks and zoonotic transmission associated with these viral agents necessitates a comprehensive understanding of their prevalence and diversity, especially in resource-limited rural settings. The swine population serves as a key mixing vessel for various emerging and re-emerging pathogens that contribute to significant economic losses on a global scale.

In developing countries, robust surveillance studies of pathogens are essential to prevent outbreaks and manage public health crises. Advances in molecular tools and high-throughput sequencing techniques, such as next-generation sequencing (NGS), mRNA expression profiling, and single nucleotide polymorphism (SNP) analysis, provide innovative ways to rapidly identify emerging pathogens in both humans and animals. The application of metagenomics to sequence the complete DNA and RNA content of samples has become crucial for identifying and characterizing novel infectious agents (Höper et al., 2017; Chiu and Miller, 2019).

Most studies on viral detection in diarrheic samples using NGS focus on identifying one virus at a time, often isolating either DNA or RNA viral genomes before preparing specific viral libraries for sequencing. However, identifying unknown viral species can be challenging due to significant differences in genomic structures and compositions.

Recent studies have exploited viral metagenomics, a method that enables the simultaneous detection of multiple viral genomes, and enhanced it with high-throughput sequencing technologies. This innovative approach has yielded significant insights into viral genetic diversity, identifying new viral species across a wide range of host organisms, environmental sources, and plant life (Thurber et al., 2009; Midgley et al., 2012; Sachsenroder et al., 2014; Chen et al., 2018; Cortey et al., 2019; Smoľak et al., 2022; Sawant et al., 2023; Qian et al., 2024). Consequently, we conducted a study to assess the diversity of the swine fecal RNA virome at a pig farm located in the Rewari district of Haryana, India. This study aims to assist researchers in evaluating the risk of disease outbreaks in Indian swine farms and inform the development of strategies to mitigate interspecies virus transmission, thereby enhancing public health safety and promoting sustainable pig farming practices.

## 2 Materials and methods

### 2.1 Collection of samples

For molecular study, a total of 15 diarrheic samples were collected from an organised pig farm of Haryana (Latitude and longitude coordinates: 28.8, 76.6) by a trained veterinarian using a standard non-invasive method. Several of animals were suffering from diarrhea with nasal and lachrymal discharges. These samples were transported to the laboratory at 4°C and stored at −80°C till further processing. All the fifteen samples from diarrheic pigs were processed for RNA extraction followed by library preparation for NGS study. The sampled animals included individuals of different age groups, ranging from 3-year-old adult pigs to 15-day-old piglets. In particular, the age distribution comprised adults (1.5–3 years), growers (9 months), and neonates (15–70 days). Complete details of sample ID, sex, age, sample type, and fecal consistency are provided in **Table 1**.

**Table 1:** Details of samples used for metagenomic study.

Sample ID	Sex	Age	Type of sample	Faecal consistency	Date of collection
ABT 106	Female	15 D	Faecal	Diarrheic	01/07/19
ABT 107	Female	15 D	Faecal	Diarrheic	
ABT 109	Female	2 Yr	Faecal	Diarrheic	
ABT 110	Female	2 Yr	Faecal	Diarrheic	
ABT 111	Female	3 Yr	Faecal	Diarrheic	
ABT 112	Female	70 D	Faecal	Diarrheic	
ABT 114	Female	70 D	Faecal	Diarrheic	
ABT 115	Female	1.5 Yr	Faecal	Diarrheic	
ABT 121	Female	9 M	Faecal	Diarrheic	
ABT 127	Female	1.5 Yr	Faecal	Diarrheic	
ABT 128	Female	2 Yr	Faecal	Diarrheic	
ABT 133	Female	2 Yr	Faecal	Diarrheic	
ABT 134	Female	2 Yr	Faecal	Loose	
ABT 139	Female	2.5 yr	Faecal	Diarrheic	
ABT 145	Female	2.5 yr	Faecal	Diarrheic	

### 2.2 Extraction of RNA

Faecal samples were resuspended in 10 volumes of phosphate-buffered saline (PBS) and vortexed briskly for 5 min. Three hundred microliters of supernatant were collected after centrifugation (5 min, 15,000 x g) and filtered through a 0.45-um filter (Millipore) to remove cell debris. Viral nucleic acids were then extracted using a combination of Trizol reagents and any viral RNA isolation kits available from various companies e.g., QIAamp Viral RNA Kit (Qiagen), which was used in these studies. Briefly, swab samples were resuspended in 400 µl of PBS. To this 400 µl of Trizol reagent was added and mixture was vortexed. To this 50 µl of DNA elimination buffer [containing Tris (pH 8.0-8.5), EDTA (0.1 mM), and DNase (20 μg/ml)] was added and the reaction was incubated at room temperature (23°C) for 15 min for removal of DNA. To this 200 µl of chloroform was added and contents were centrifuged at 12,000 rpm for 10 minutes. The aqueous phase containing nucleic acid was taken and to this an equal volume of isopropanol was added. This mixture was then transferred to column that comes with QIAamp Viral RNA Kit (Qiagen). The columns were centrifuged at 10,000 rpm for 1 min. The flow through was discarded. The columns were then washed with 500 µl of AW1 solution and 500 µl of AW2 solution successively by centrifugation at 12,000 rpm for 1 min. The viral RNA was eluted in 30µl of nuclease free water. The extracted RNA was quantified using a Qubit^®^ 2.0 Fluorometer (Invitrogen). The purity and integrity of RNA were also checked with an AATI Fragment Analyzer (Agilent Technologies, USA).

### 2.3 cDNA synthesis

Purified RNAs were used for cDNA synthesis using random hexamersas described previously (Maan et al., 2019). Briefly, 0.4 μM of a random hexamer primers were used in a reverse transcription reaction with 1 μl of SuperScript III reverse transcriptase (Invitrogen)(200 units/μl) and 50ng/μl of RNA. Second strand of cDNA synthesis was then performed using exo-Klenow fragment polymerase (New England Bio Labs). The cDNA samples were then purified using AMPureXP magnetic beads as per manufacturer’s instructions followed by quantitation of purified cDNA with Qubit 2.0 fluorometer. As the samples were collected from same pig farm, all fifteen cDNAs were pooled as one sample by mixing equimolar amounts of each cDNA.

### 2.4 cDNA library preparation and whole genome sequencing

**Figure 1:**
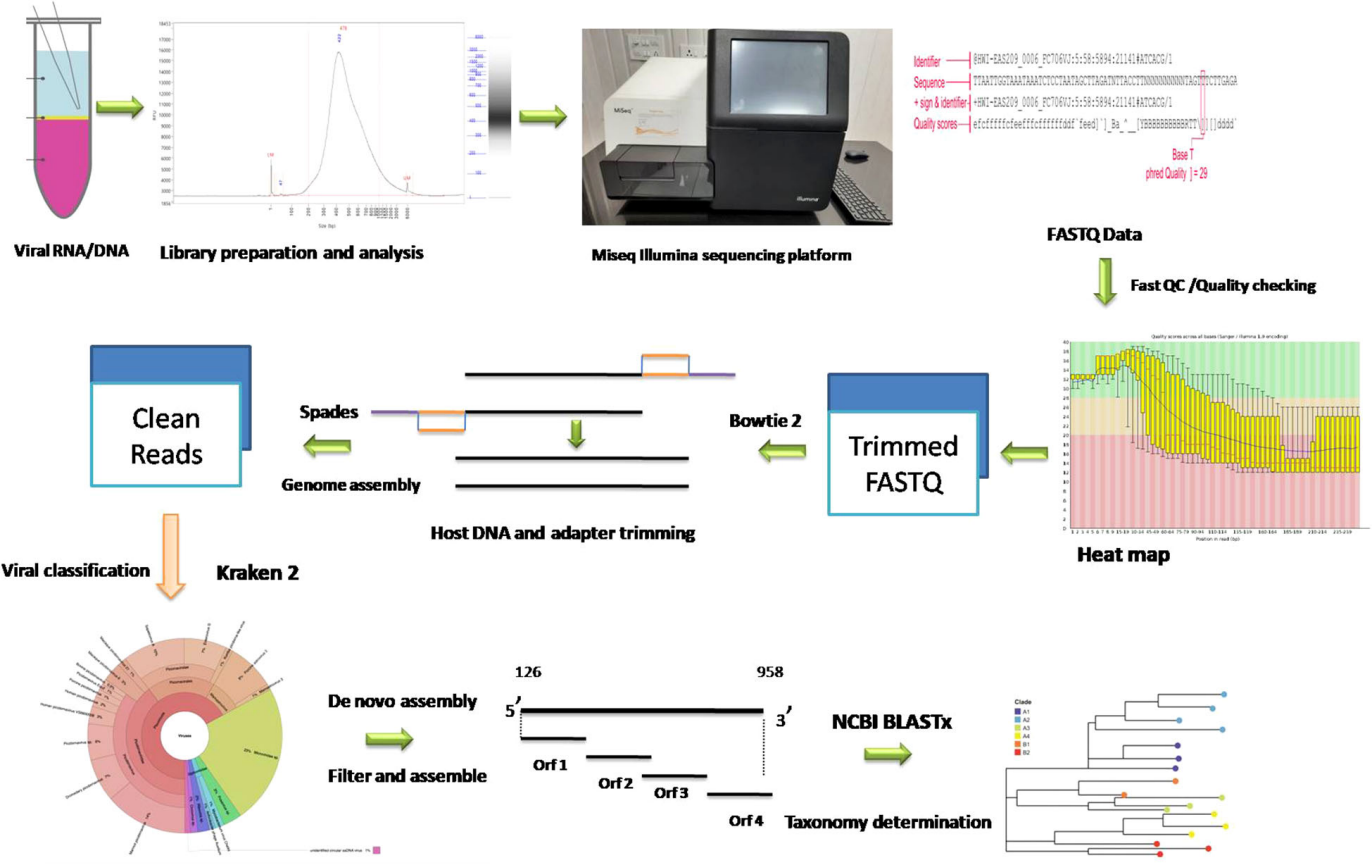
Schematic diagram of metagenomic study conducted on porcine samples.

The cDNA library was prepared using Nextera XT DNA Library Prep Kit using the standard protocol. The purity and integrity of cDNA library prepared was checked with an AATI Fragment Analyser (Agilent Technologies, USA). A 300-cycle, 150 bp paired-end sequencing protocol was used for sequencing on an Illumina MiSeq instrument as recommended by the manufacturer. The overview of workflow for metagenomic characterization of porcine faecal virome is depicted in **Figure 1**.

### 2.5 Data assembly and processing

The FASTQ files were then subjected to quality checking using FastQC, version 0.12.1 (Andrews, 2010). The sequences were then subjected to trimming to remove Illumina adapters, low-quality reads, and primers using TRIMMOMATIC, version 0.39, with a minimum quality score of 20 (Bolger et al., 2014). The output files were then again subjected to quality checking using FastQC. Host contamination was removed using Bowtie2, version 2.4.4 (Langmead and Salzberg, 2012). The SPAdes version 3.15.2 (Prjibelski et al., 2020) with kmer values of 21, 31, 41, 51, 61, and 71, was used to assemble reads with the option to minimize the number of mismatches in the final contigs. The cleaned reads were classified using the Kraken viral 2020 database with Kraken version 1.3.1 (Wood and Salzberg, 2014) and default parameters on the Galaxy server. Classifying cleaned reads was a deliberate methodological choice that enhanced the overall accuracy and interpretability of the contig-based results. The Krona pie chart from the taxonomic profile tool was used to render the results of our metagenomic profiling (Ondov et al., 2011). The virus reads were then extracted and assembled using SPAdes, and the resulting contigs were analyzed using BLASTx at NCBI to determine taxonomy (Altschul et al., 1997). ORFs of assembled contig/genome were predicted using Vgas tool with default parameters (Zhang et al., 2019).

### 2.6 Sequence and phylogenetic analysis

The contigs obtained from NGS data were analysed using the BLAST tool available at the NCBI [http://www.ncbi.nlm.nih.gov/BLAST/]. The final contig sequences of each viral genome were aligned using the program MUSCLE, version 5 (Edgar, 2004), with the published nucleotide sequences of corresponding viruses obtained from GenBank. MEGA, version 7 (Kumar et al., 2016), software was used to conduct phylogenetic analyses using the Neighbor-joining [NJ] algorithm. The AA/NT identities were calculated using distance matrix method implied in MEGA software. For visualization purposes, iTOL, version 5 (Letunic and Bork, 2021), an interactive Tree of Life, was employed. All the complete and important partial genomes extracted from the viral metagenome were considered for phylogeny construction and analysis, and five hundred bootstrap replications of the data were run to determine the robustness of tree branching. All four predicted complete genomes were visualized, and ORFs were mapped using the Proksee visualization tool (Grant et al., 2023).

Nucleotide sequence accession number: Sequence reads were deposited in genbank under the biosample accession number: SAMN37033531.

## 3 Results

### 3.1 Overview of sequence data

**Figure 2:**
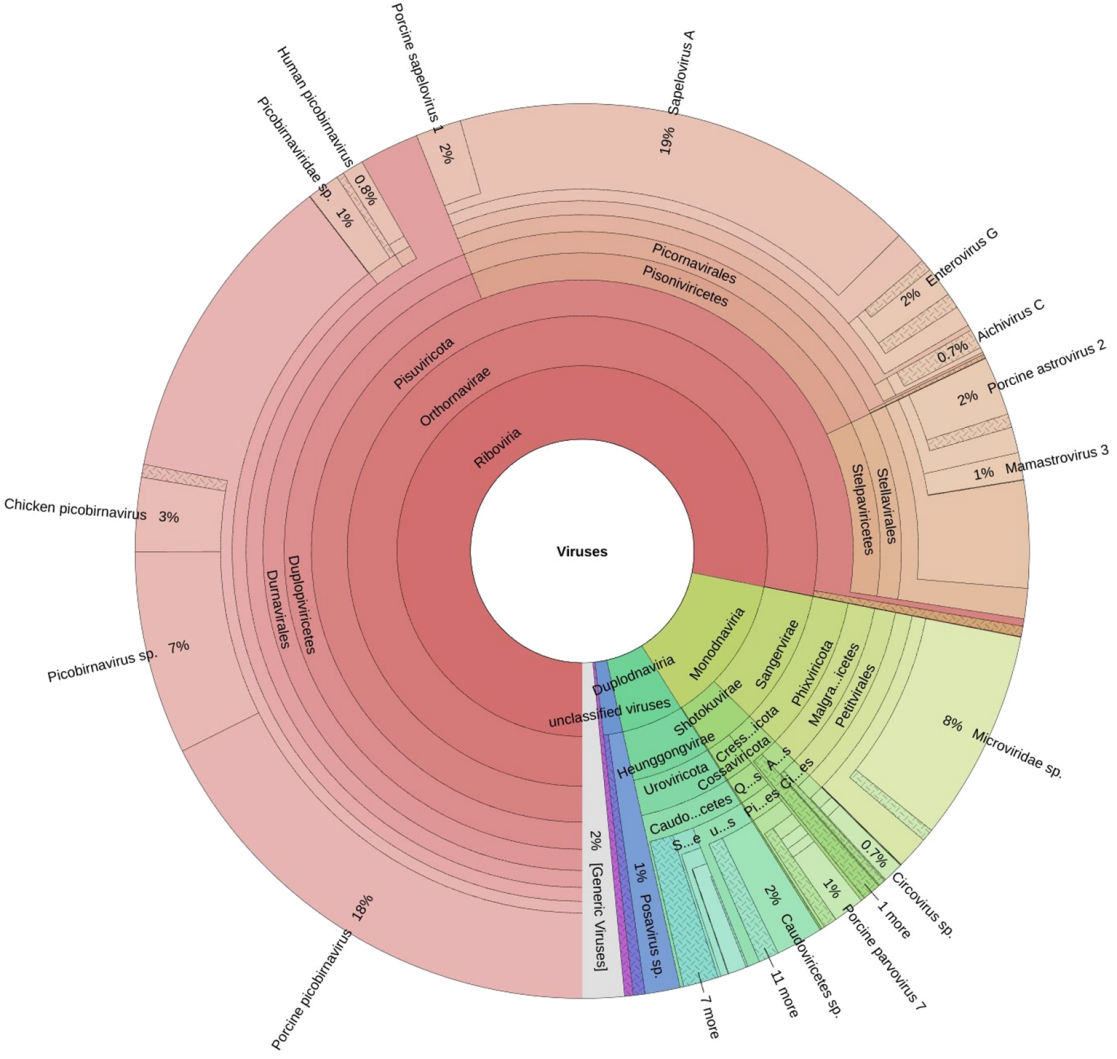
The Krona plot shows taxonomically classified detected reads and reported viruses as pie charts.

**Figure 3:**
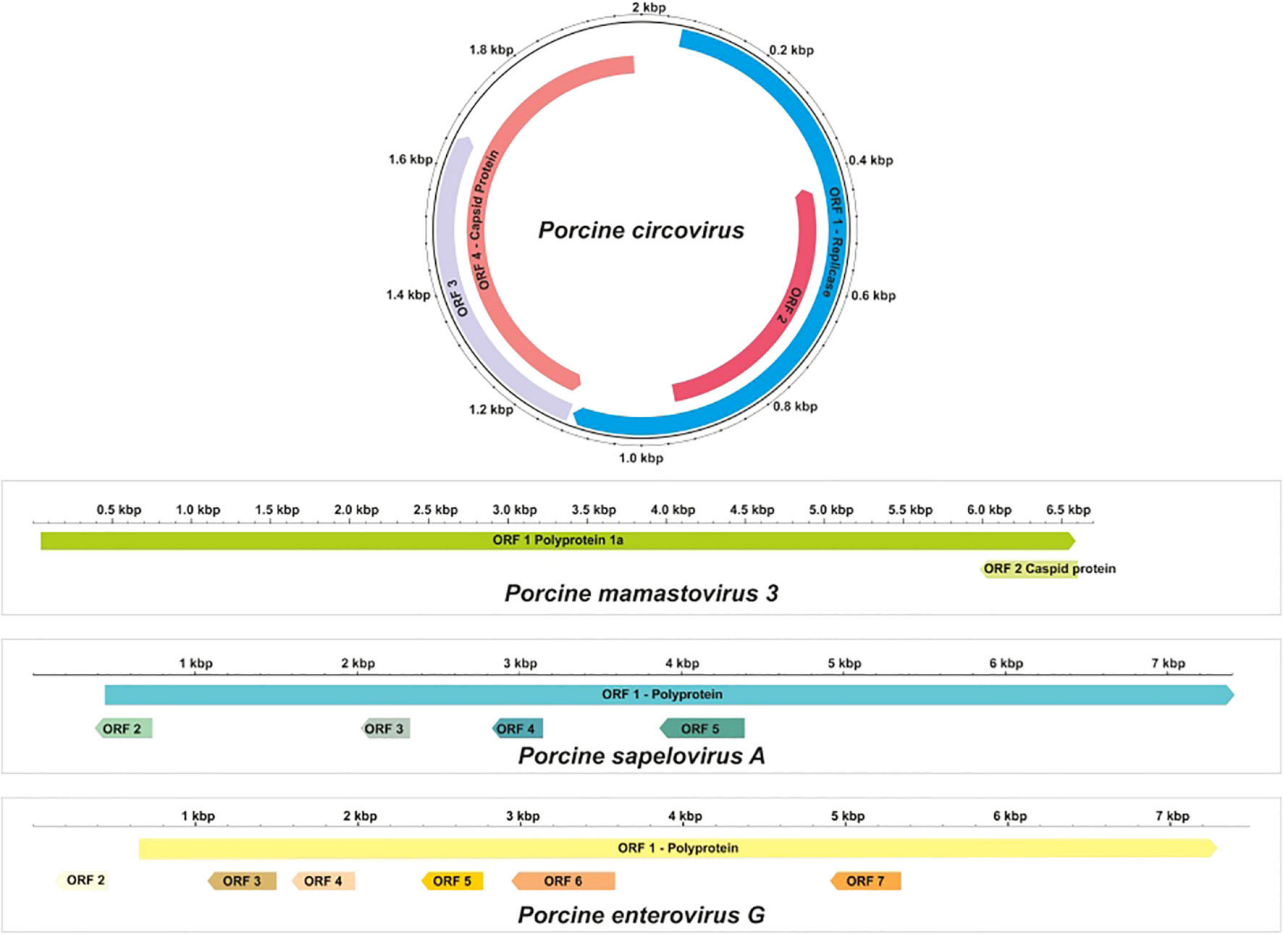
Genome structure of most abundant viruses found during metagenomic analysis.

**Figure 4:**
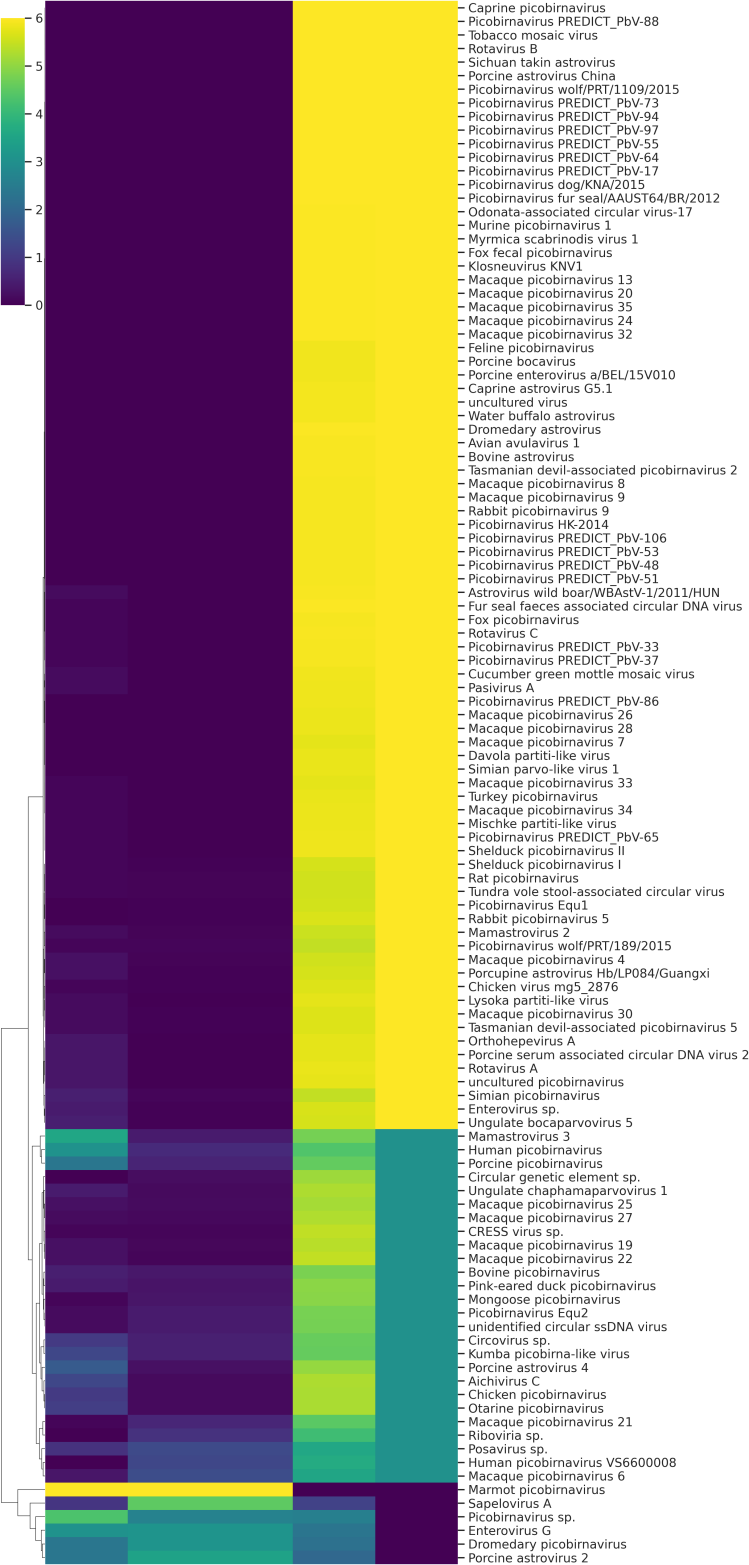
Heat map of different viruses detected during metagenomic analysis.

A total of fifteen faecal samples from pigs showing symptoms of diarrhea and nasal discharge were collected from high density pig farm. Viral nucleic acids were enriched by filtration and nuclease treatment prior to nucleic acid extraction, random RT-PCR-based amplification, and illumina sequencing. A total of 2653974 sequences and 343.3Mbp bases were present with sequence lengths in the range of 35-150 bp. A total of 7469 sequences were trimmed and removed, and the Trimmomatic analysis resulted in a total of 2646505 sequences with 343.1 Mbp bases. Sequence contigs were generated using reads from pooled sample and classified based on best BLASTx expectation (E) scores. 19.51 percent of all the sequence reads had no significant similarity to any sequences in the GenBank. The most abundant fraction of viral sequences showed matches to mammalian viruses (**Table 2**; **Figures 2**–**4**). BLAST analysis revealed the closest homologs and their associated host species. Most of them showed the closest identity with viruses of porcine origin (*Sus scrofa*). An exception was the capsid protein segment of *Porcine picobirnavirus*, which showed closest identity to a PBV strain isolated from *Macaca mulatta* (**Supplementary Table 1**).

**Table 2:** Relative number of viral families represented in the pooled porcine fecal metagenome.

Sl. no.	Virus family	Family ID	Relative no. of reads	Relative percentages
1	*Picobirnaviridae*	585893	7083	59.61%
2	*Picornaviridae*	12058	2860	24.07%
3	*Astroviridae*	39733	1555	13.09%
4	Unknown	–	1305	10.98%
5	*Circoviridae*	39724	191	1.61%
6	*Parvoviridae*	10780	78	0.66%
7	*Reoviridae*	10880	8	0.07%
8	*Hepeviridae*	291484	7	0.06%
9	*Mimiviridae*	549779	1	0.01%
10	*Paramyxoviridae*	11158	1	0.01%
11	*Partitiviridae*	11012	10	0.08%
12	*Polycipiviridae*	2169570	1	0.01%
13	*Virgaviridae*	675071	5	0.04%

### 3.2 Fecal virome

A remarkably high level of enteric infection was detected, involving both known and previously unreported viral species, indicating significant viral transmission among these animals. Sequence reads with the highest BLASTx scores matched mammalian RNA viruses from the families *Picobirnaviridae, Picornaviridae, Astroviridae, Parvoviridae, Partitiviridae, Reoviridae, Hepeviridae, Virgaviridae, Paramyxoviridae, Polycipiviridae*, as well as DNA viruses from the *Circoviridae* and *Mimiviridae*. Previously characterized viruses identified in this study included kobuviruses, enteroviruses, sapeloviruses, picobirnavirus, and porcine circovirus (**Figure 2**). The number of viral reads in the pooled sample was sufficiently high to enable the detection of unknown viral taxa. However, several viruses, including picobirnavirus, posavirus 1, kobuvirus, rotavirus, parvovirus, klosneuvirus (KNV1), and swine pasivirus (SPaV), were present at relatively low read counts in the fecal sample (<1000 RPM of raw read pairs in the dataset). Additionally, low read counts were observed for sequences resembling unclassified viruses previously documented in fecal samples from various domestic animals.

#### 3.2.1 Picornaviruses

The order *Picornavirales* comprises a diverse group of viruses that infect a wide range of hosts. The genome is monopartite and its length varies between 7.2 and 9.8 kb (Oude Munnink et al., 2017). In this study, genomes from four genera—*Sapelovirus, Enterovirus, Kobuvirus*, and *Posavirus* were identified in pooled samples, comprising 2,592 reads.

##### 3.2.1.1 Porcine sapelovirus A

*Porcine Sapelovirus* A (PSV-A) was present at a high load in the fecal samples, enabling the *de novo* assembly of its complete genome sequence. The assembled genome consisted of a 7,422-nucleotide (nt) contig with 1,495 reads, achieving an average positional coverage of 94.6%. The complete genome of PSV-A has been submitted to GenBank under accession number SAMN37033531.

**Figure 5:**
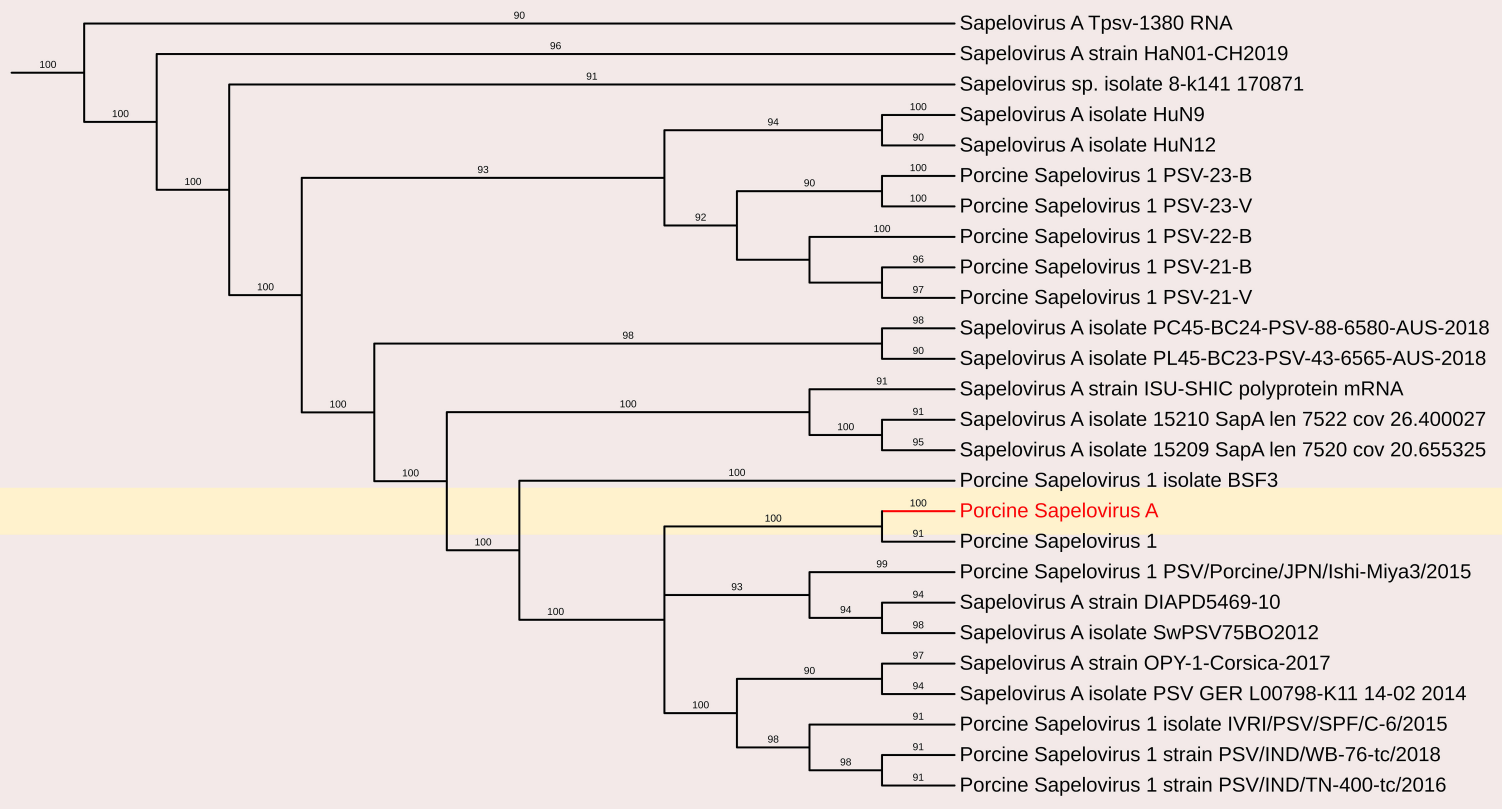
The phylogenetic tree displaying relationships between different porcine Sapelovirus based on complete genome sequences. The neighbour-joining tree was established based on the complete nucleotide sequences. The bar represents the genetic distance while numbers indicate the bootstrap replicates.

Phylogenetic analysis was conducted by comparing the whole genome sequence of PSV-A with other globally circulating strains. The sequencing results revealed an open reading frame (ORF) encoding a polyprotein of 2,331 amino acids (aa), along with four additional undefined ORFs. Whole-genome analysis showed a maximum nucleotide identity of 88.4% to an Indian strain of PSV-A 1 isolate IVRI/PSV/SPF (accession no. KY053835), 86.13% similarity with American strain and a minimum of 83.8% nt identity to the South Korean isolate KU2022PSV01 (accession no. OQ722357) (**Figures 3**, **5**).

Porcine Sapelovirus consists of a single serotype, is restricted to pigs, and is not known to infect humans. Its genome contains a single ORF encoding a polyprotein that is cleaved into 12 mature structural and functional proteins: a leader protein (L), four structural proteins (VP1–VP4), and seven nonstructural proteins (2A–2C, 3A–3D) (Chelli et al., 2020).

##### 3.2.1.2 Porcine enterovirus G

**Figure 6:**
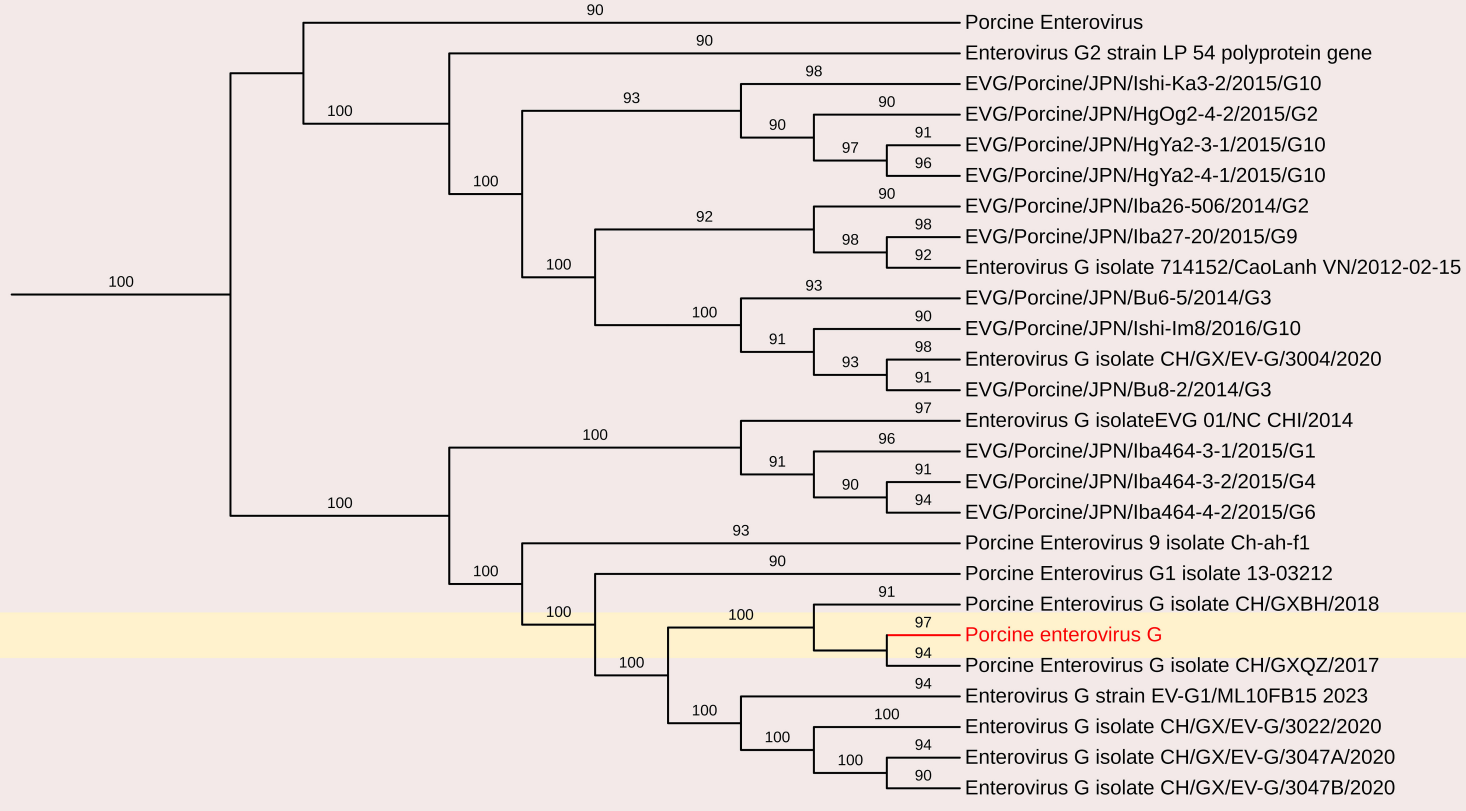
The phylogenetic tree displaying relationships between different porcine Enterovirus G based on complete genome sequences. The neighbour-joining tree was established based on the complete nucleotide sequences. The bar represents the genetic distance while numbers indicate the bootstrap replicates.

The complete coding genome sequence of a Porcine enterovirus G (PEV-G) was obtained from the sample. The assembled genome consisted of a 6191 nt contig containing 1042 reads, achieving an average positional coverage of 96.2%. The complete genome of *Porcine Sapelovirus* has been submitted to GenBank under accession number SAMN37033531. The genome region analysis revealed highest nt identity of 100% with PSV-A isolate CH/GXQZ/2017 from China (Accession no. MT274669). It showed minimum 80.90% nt identity to Enterovirus G strain EVG/Porcine/JPN/Bu5-6/2014/G3 from Japan (Accession no. LC316804) (**Figures 3**, **6**). The PSV-A includes seven ORFs in which a single open reading frame (ORF 1), encodes for four structural proteins (VP4, VP2, VP3, VP1), and seven non-structural proteins (2A–C, 3A–D).

##### 3.2.1.3 Kobuvirus

A partial sequence of Porcine kobuvirus (PoKV) (589 nt; 25.8–54.8% genome coverage) was obtained, which was closely related (90.6% nt identity) to the kobuvirus strain (PoKoV/Iba444-2/JPN/2016) reported from Japan (Acc. No. LC210613) and minimum 85.5% nt sequence identity to kobuvirus strain (KobuV/Pig-wt/ESP/B304/2017) from Spain (Accession No. MK962328).

##### 3.2.1.4 Porcine stool-associated RNA virus (Porcine posavirus)

*Porcine stool-associated RNA virus* (posavirus) have been detected in the feces of healthy pigs and in water collected from swine farms (Shan et al., 2011; Hause et al., 2015; Hause et al., 2016). Similarly, fish stool-associated RNA virus (fisavirus) was identified in the intestinal content of a healthy carp (Reuter et al., 2015), and human stool-associated RNA virus (husavirus) was found in the feces of predominantly healthy individuals (Oude Munnink et al., 2015). Although these viruses share structural similarities based on genome organization, they exhibit broad genetic diversity, often showing less than 40% amino acid identity in specific coding regions. This suggests a deep evolutionary history within this virus family.

**Figure 7:**
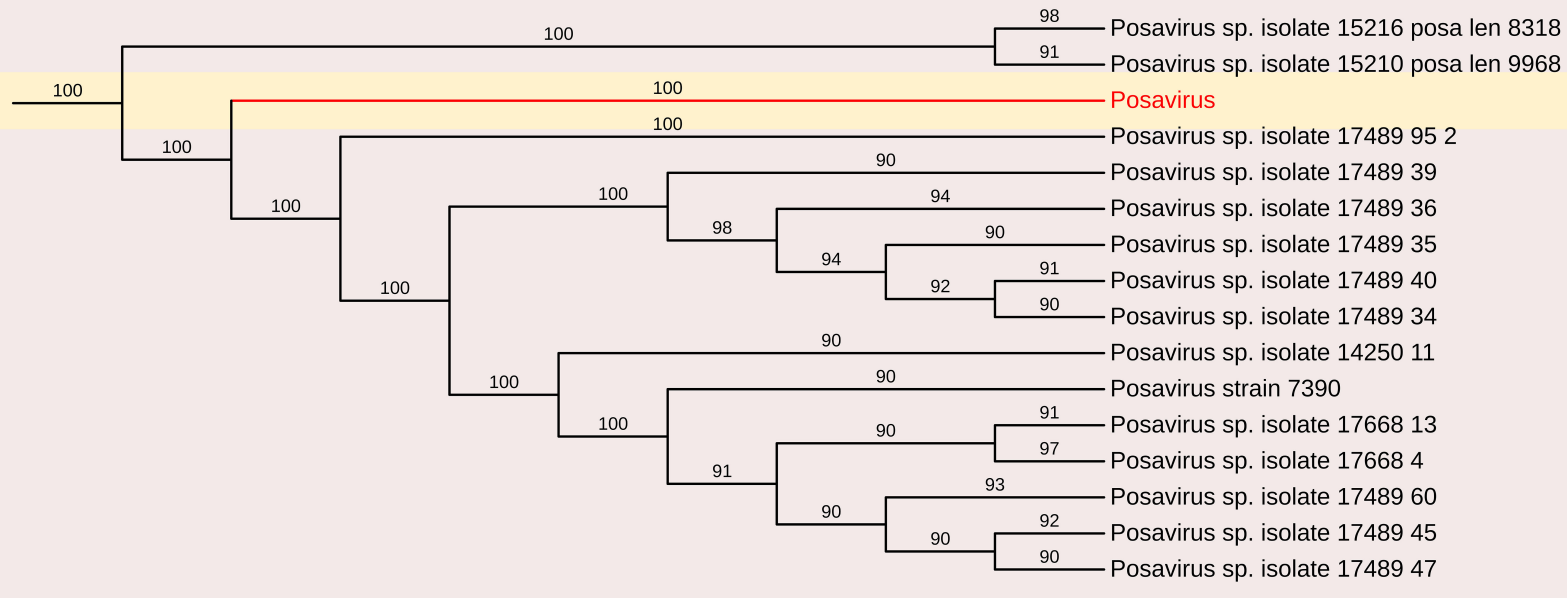
The phylogenetic tree displaying relationships between different porcine Posavirus-based on partial genome sequences. The neighbour-joining tree was established based on the complete nucleotide sequences. The bar represents the genetic distance while numbers indicate the bootstrap replicates.

A partial genome sequence of posavirus was obtained from the sample, consisting of a 2,109 nt contig with 172 reads and an average positional coverage of 98.8% (GenBank accession number SAMN37033531). Sequence analysis revealed a maximum nucleotide identity of 100% with posavirus 1 strain 9470 from the USA (Accession no. KT833062) and posavirus 1 strain HBTS-11 from China (Accession no. KU981058). The lowest nt identity (81.3%) was observed with posavirus 1 strain 8295 from North Carolina, USA (Accession no. KT833065)(**Figure 7**).

#### 3.2.2 Porcine astrovirus

The *Astroviridae* family comprises positive-sense single-stranded RNA (ssRNA) viruses with genome sizes ranging from 6.4 to 7.3 kb. Astroviruses (AstV) are known to cause gastroenteritis in both mammalian and avian species.

**Figure 8:**
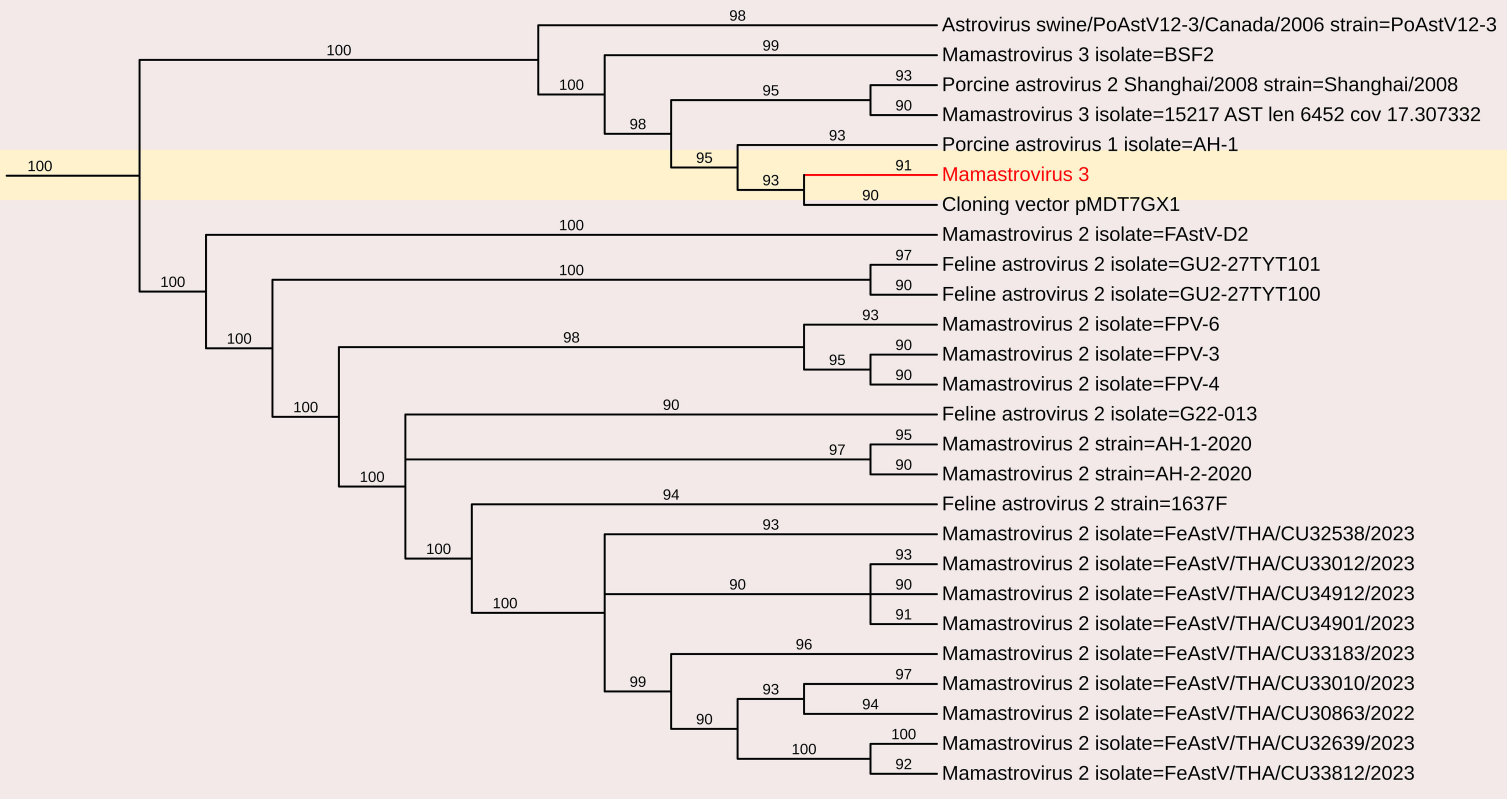
The phylogenetic tree displaying relationships between different porcine Mamastrovirus 3 based on complete genome sequences. The neighbour-joining tree was established based on the complete nucleotide sequences. The bar represents the genetic distance while numbers indicate the bootstrap replicates.

In this study, astrovirus sequences were detected in porcine fecal samples. *De novo* assembly of the complete genome sequence of porcine mamastrovirus 3 was obtained from a fecal sample, yielding a 6,196 nt contig with 1,391 reads and an average positional coverage of 95%. The virus genome contains two ORFs encoding a polyprotein of 6,489 amino acids (aa) in ORF1 and 621 aa in ORF2. Phylogenetic analysis was conducted by comparing the whole genome sequence with other globally circulating strains. Whole-genome analysis revealed a maximum nt identity of 86.3% with mamastrovirus 3 isolate PAstV-GX1 from China (Accession no. KF787112). It shared 77.9% nt identity with mamastrovirus 2 isolate FeAstV/THA/CU33183/2023 from Thailand, while the lowest identity (73.3%) was observed with mamastrovirus 3 isolate 15209 from the USA (Accession no. MW504556)(**Figures 3**, **8**).

#### 3.2.3 Porcine circovirus 3

Porcine circoviruses (PCVs) are small, non-enveloped, single-stranded DNA viruses belonging to the family *Circoviridae*. PCV consists of four distinct species: PCV1, PCV2, PCV3, and PCV4, each exhibiting significant differences that lead to limited cross-protection among them. PCV2 is responsible for causing considerable economic losses within the swine industry. PCV3 is suspected of having a comparable impact to PCV2.

**Figure 9:**
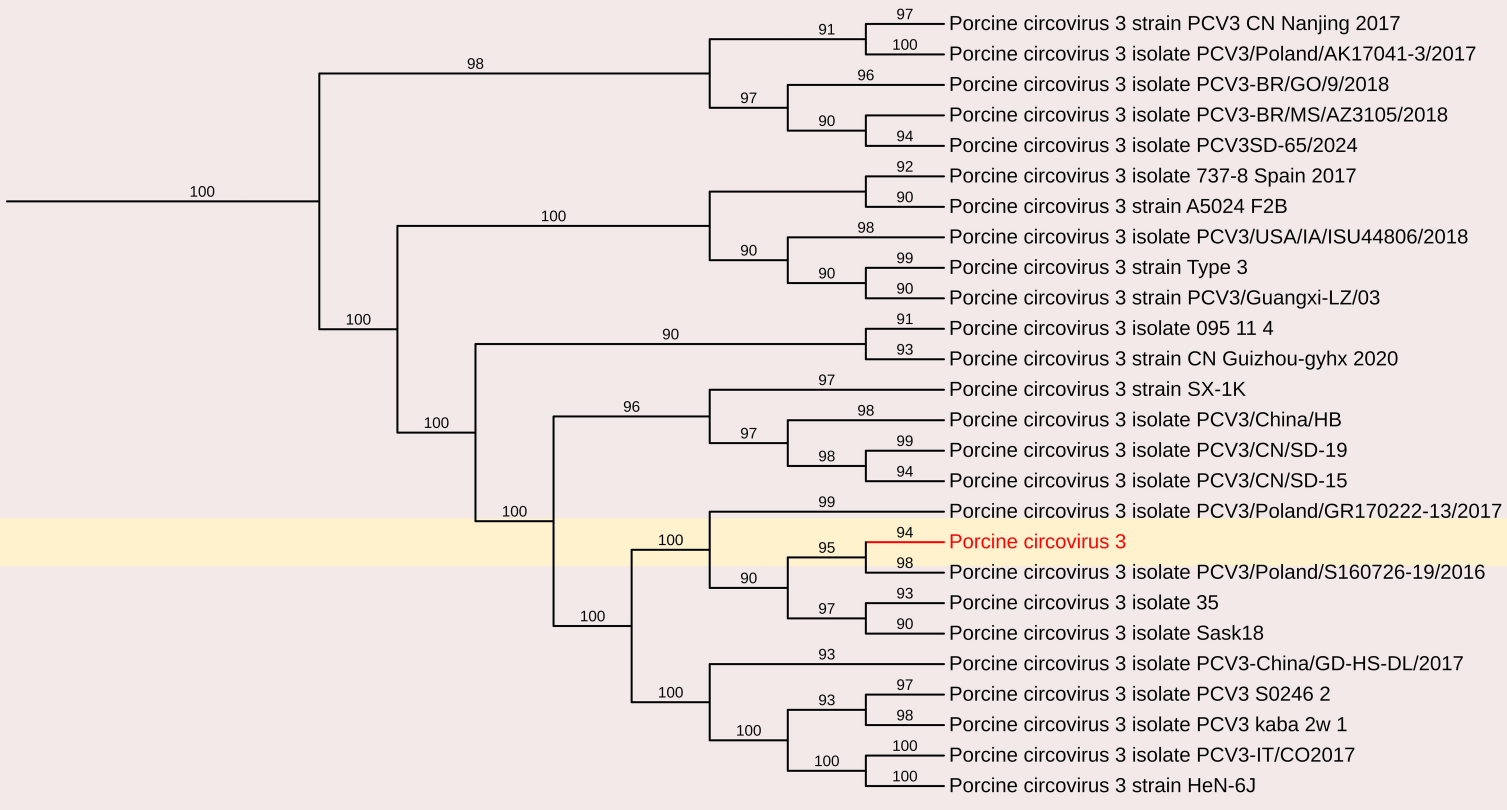
The phylogenetic tree displaying relationships between different porcine circovirus 3-based on complete genome sequences. The neighbour-joining tree was established based on the complete nucleotide sequences. The bar represents the genetic distance while numbers indicate the bootstrap replicates.

The complete genome sequence of PCV was obtained from the fecal sample, yielding a 2003 nt contig with 172 reads and an average positional coverage of 98.8%. The coding sequence of this PCV strain has been deposited in GenBank under Accession Number SAMN37033531. Phylogenetic analysis was performed by comparing the genome sequence of this strain with other globally circulating PCV strains. Whole-genome analysis revealed a maximum nt identity of 100% with PCV isolates PCV3/Pig/CN/ShanXi170709, PCV3/FJ37, and PCV3 from China (Accession Nos. MF769811, MN075133, and MG870097, respectively). The lowest nt identity (99.6%) was observed with Porcine Circovirus 3 strain HeN-7C from China (Accession No. PP067102) (**Figures 3**, **9**).

### 3.3 Other partial genome sequences

#### 3.3.1 Porcine parvovirus 7

**Figure 10:**
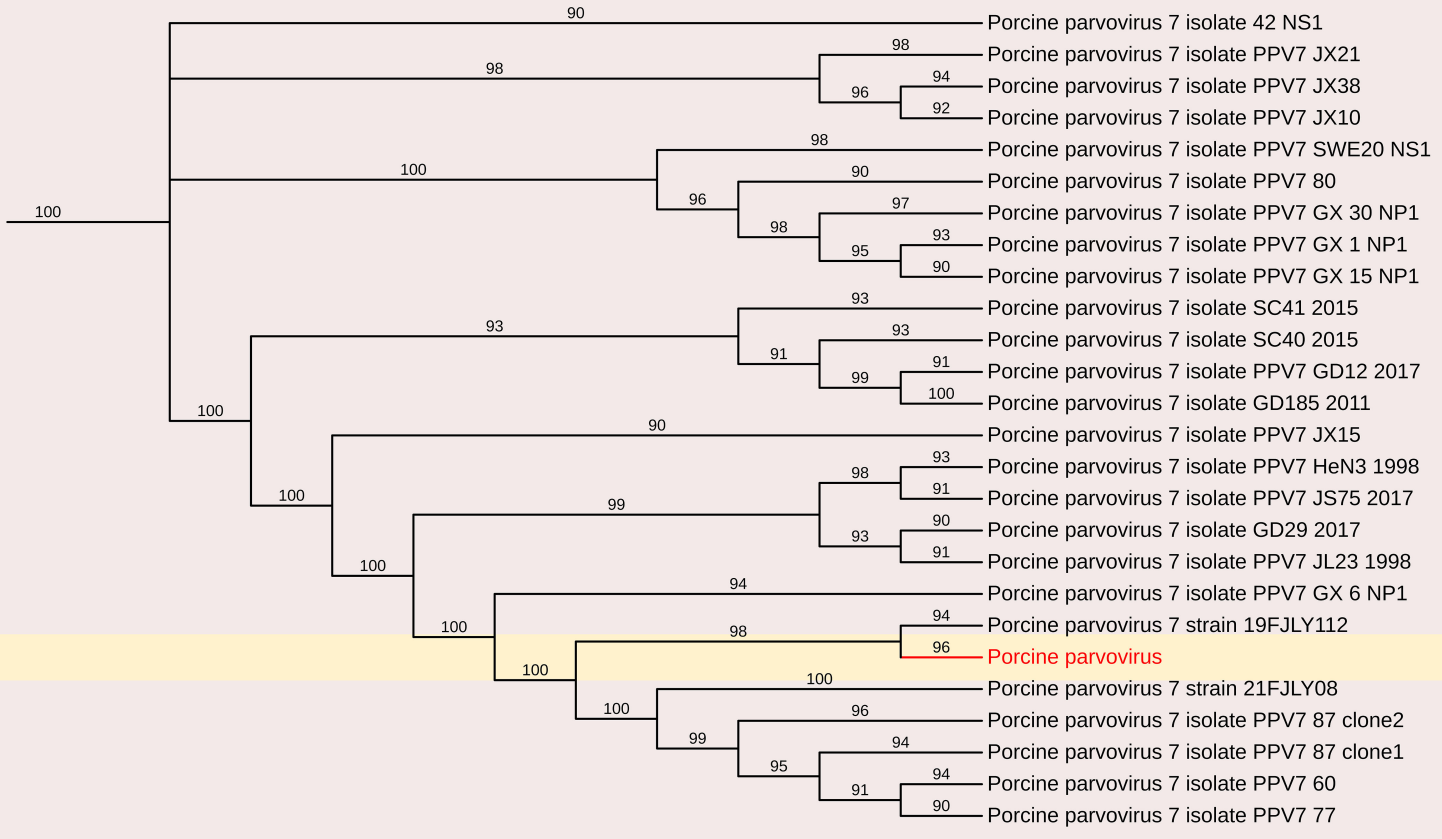
The phylogenetic tree displaying relationships between different porcine Parvovirus 7-based on partial capsid gene sequences. The neighbour-joining tree was established based on the complete nucleotide sequences. The bar represents the genetic distance while numbers indicate the bootstrap replicates.

Parvoviruses constitute a diverse group of viruses that infect a wide range of animals and humans by causing disease on their own or in association with other viruses, for example adenoviruses and picobirnaviruses. In this study, partial sequences of PPV7 were obtained and characterized. Analysis of a 701 nt sequence (3023bp to 3724bp in PPV7 isolate GX49, complete genome) revealed a maximum nt identity of 96.3% with the Chinese PPV7 isolate GX14-1998, partial genome and PPV7 strain 21FJ13, complete genome (Accession Nos. MN326253 and ON462335). However, it exhibited a lower nt identity of 92.3% with the Chinese PPV7 isolate PPV7-CS-3 and PPV7 strain AH-PPV720178-1 (Accession Nos. MZ803089 and MW853958). Additionally, characterization of an 857-nt sequence (2876 bp to 3733 bp in PPV7 isolate GX49, complete genome) of PPV7 obtained in this study showed a maximum nt identity of 96.1% with the Chinese strain 20FJSM34 (Accession No. OQ983812). In contrast, it demonstrated a lower nt identity of 89.7% with the other Chinese PPV7 isolate PPV7-XZ7-1999 and PPV7 isolate GD60-2011 (Accession Nos. MN326252 and MN326248)(**Figure 10**).

#### 3.3.2 Porcine rotavirus

Rotaviruses (RVs) are a leading cause of acute viral gastroenteritis in young animals and children worldwide. Among the nine recognized RV genogroups (A–I), *Rotavirus* A (RVA), *Rotavirus* B (RVB), and *Rotavirus* C (RVC) are primarily associated with diarrhea in piglets. In this study, several short contigs were identified, resembling VP1, VP2, VP3, and NSP2 of *Porcine Rotavirus* A and VP2, NSP2, NSP3, and VP7 of *Porcine Rotavirus* C. These sequences were too short to draw any phylogeny based conclusions but these findings highlight the potential role of rotaviruses as important enteric pathogens in piglets, emphasizing the need to include them in routine differential diagnoses of enteric diseases in swine.

#### 3.3.3 Picobirnavirus

Picobirnaviruses (PBVs) are widely found in mammalian feces and have been classified under the *Picobirnaviridae* family by the International Committee on Taxonomy of Viruses (ICTV). These viruses are small, highly variable, non-enveloped, double-stranded RNA (dsRNA) viruses with a bi-segmented genome (Ganesh et al., 2012; Mondal et al., 2013). The larger genomic segment, segment 1 (L), ranges from 2.2 to 2.7 kb and encodes the capsid protein, along with an additional open reading frame that encodes a putative protein of unknown function. The smaller genomic segment, segment 2 (S), is between 1.2 and 1.9 kb and encodes the RNA-dependent RNA polymerase (RdRp).

PBVs are classified into two primary genogroups based on the sequence of the RdRp gene: Genogroup I, represented by the Chinese strain 1-CHN-97 (AF246939) and Genogroup II, represented by the US strain 4-GA-91 (AF246940) (Woo et al., 2016; Rosen et al., 2000; Bhattacharya et al., 2007; Ganesh et al., 2012). Recent studies have also reported the detection of novel PBV genogroups in human and environmental samples, suggesting their potential role as opportunistic enteric pathogens (Delmas et al., 2019; Giordano et al., 1998; Malik et al., 2014b; Verma et al., 2015; Woo et al., 2019).

##### 3.3.3.1 Porcine PBVs segments 1 and 2 sequence analysis

A total of 368 contigs belonging to *Picobirnavirus* were assembled. Among these, three RNA-dependent RNA polymerase (RdRp) segments and six capsid/open reading frame (ORF) segments greater than 1 kb were identified from pooled porcine samples.

###### 3.3.3.1.1 Genomic analysis

Segment 1 (Capsid/ORF): Six sequences were analyzed, ranging in length from 1,505 to 2,618 bases (Near-complete or partial sequences), with a G+C content of 35.28% to 46.37%. Each sequence contained a single long ORF encoding the capsid protein, which consisted of 361–537 amino acids.

Segment 2 (RdRp): Three sequences were identified, with lengths of 1,093, 1,517, and 1,870 bases, and a G+C content ranging from 42.29% to 46.14%. Each sequence contained a single long ORF encoding the RdRp protein, comprising 285–469 amino acids.

##### 3.3.3.2 Phylogenetic and comparative nucleotide analysis

The nucleotide sequences and deduced amino acid sequences of both genome segments were compared with previously reported PBV strains. Phylogenetic analysis of all capsid and RdRp segments confirmed high genetic diversity within the genus, with sequences clustering into genogroups I and II. The genotypes GI, GII, and GIII formed distinct branches, consistent with previous studies (Ganesh et al., 2011; Gillman et al., 2013; Chen et al., 2014; Smits et al., 2014; Masachessi et al., 2015). The RdRp sequences shared 31.3–95.1% nt identity with other genogroup I PBV strains, showing the highest nt identity to PPBV isolate 15217 from swine slurry in a North American swine farm (Accession No. MW977212).

The nucleotide sequences encoding the capsid protein exhibited low identity (28.4–50.8%) with other PBV strains. Phylogenetic analysis revealed the highest nt identity to Turkey PBV USA/2012/Equine/Equ2 (GenBank No. KR902506), Dubai/2013/Dromedary/GpI-2 (GenBank No. LC337999), and Human PBV Colombia/2019/Human/NYB4138 (Accession No. OL875312).

##### 3.3.3.3 Protein sequence comparisons and phylogenetic analysis

**Figure 11:**
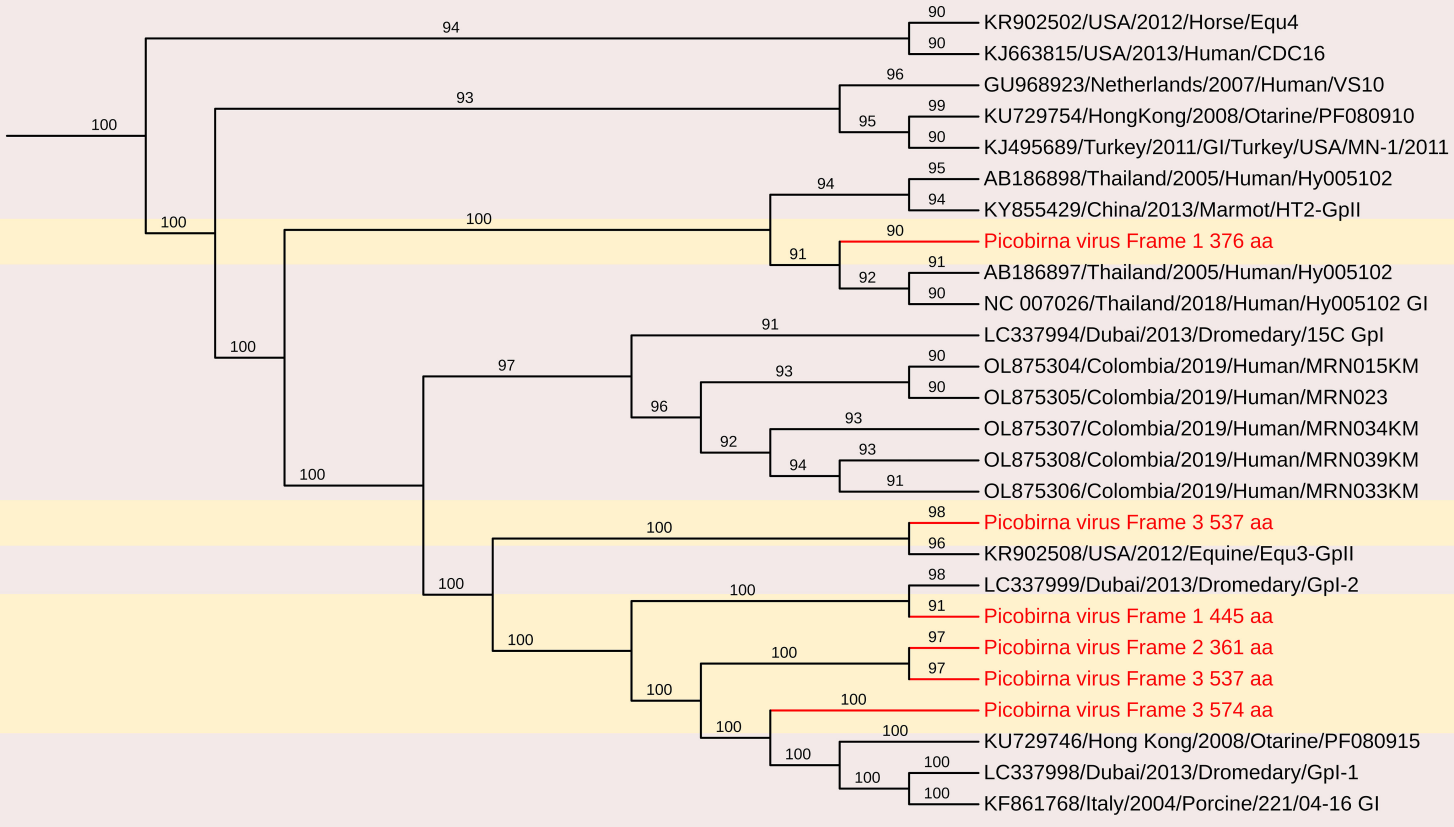
The phylogenetic tree displaying relationships between different porcine Picobirnaviruses based on amino acid sequences of capsid gene. The neighbour-joining tree was established based on the complete nucleotide sequences. The bar represents the genetic distance while numbers indicate the bootstrap replicates.

The deduced amino acid sequences of the capsid gene showed a wide range of identity levels with other PBV strains, with the closest relationships observed with:

- 87.87% Macaque PBV 6 isolate ‘WUSTL’ (USA) – (GenBank Accession No. AVD54034)
- 65% aa identity with HPBV isolate ‘ctpjD’ (USA) – (GenBank Accession No. DAG68949)
- 51.56% aa identity with Marmot PBV isolate clone c325665 (China) – (GenBank Accession No. AVX53819)
- 38% aa identity with BPBV isolate MLZ_ct74 (China) – (GenBank Accession No. UUA79503)
- 34.23% aa identity with Marmot PBV isolate clone c325708 (China) – (GenBank Accession No. AVX53573)
- 31.11% aa identity with Marmot PBV isolate clone c333175 (China) – (GenBank Accession No. AVX53629)(**Figure 11**).

Upstream to the ORF for the capsid protein, there were one to two short ORFs in the segment 1, consistent with the organization of the segment 1 in other known PBVs (Wakuda et al., 2005; Bodewes et al., 2013; Bányai et al., 2014). These findings highlight the genetic diversity of PBV capsid sequences, reinforcing the need for further studies on their evolutionary relationships and potential host adaptations.

**Figure 12:**
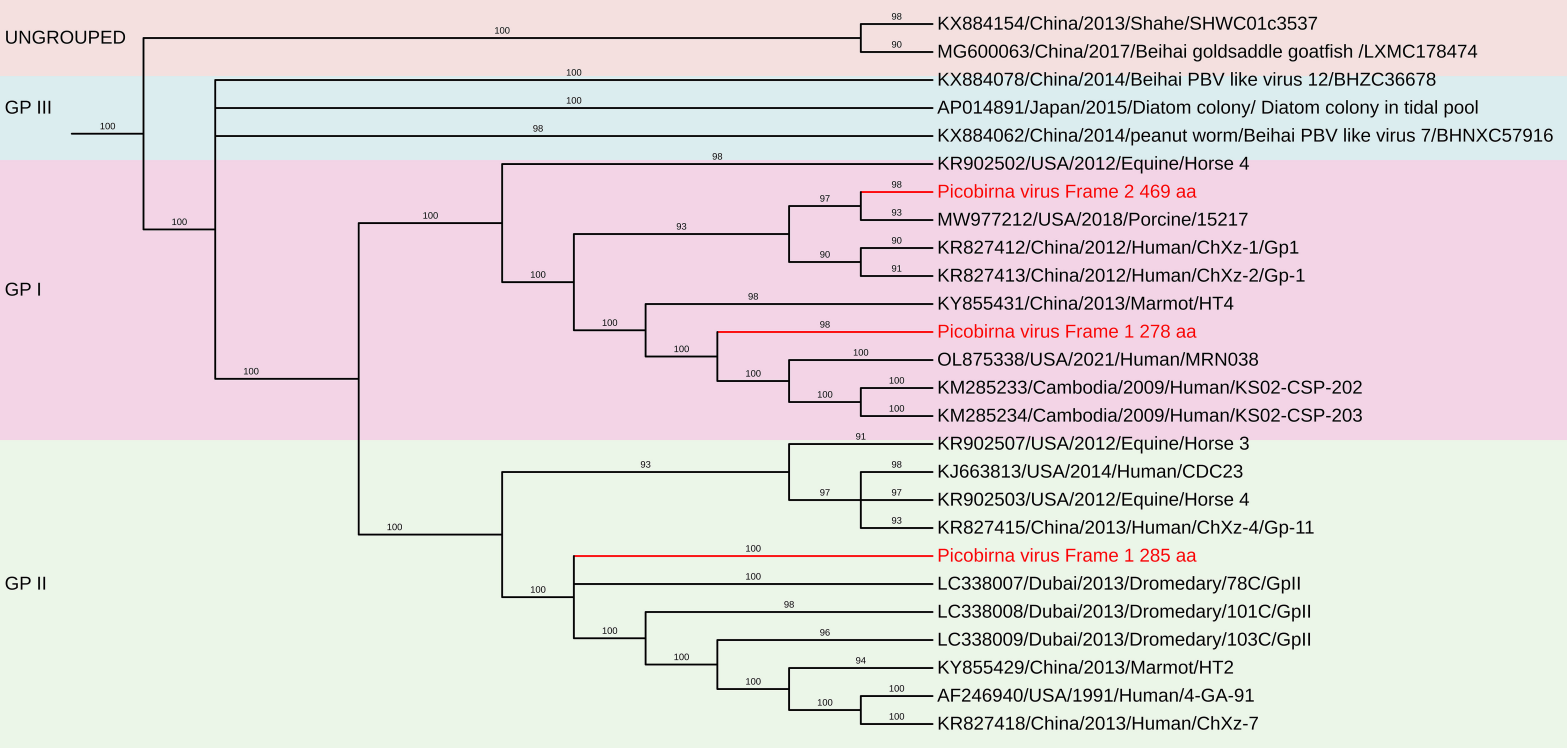
The phylogenetic tree displaying relationships between different porcine Picobirnaviruses based on amino acid sequences of RdRp gene. The neighbour-joining tree was established based on the complete nucleotide sequences. The bar represents the genetic distance while numbers indicate the bootstrap replicates.

The deduced RdRp protein sequences exhibited high amino acid identity with other PBV strains, closely related to PPBV isolate 364R-k141_162232 from China (Accession No. UDL14475 – 99.65%), PPBV isolate 15217 from a North American swine farm (Accession No. UAW00506 – 99.57%), PBV isolate 274-k141_96360 from China (Accession No. UDL14604 – 96.76%)(**Figure 12**). These findings reinforce the genetic diversity of PBV and its widespread presence in swine populations, emphasizing the need for continued surveillance and characterization. These sequences contained the conserved GDD motif, a hallmark of RdRp in dsRNA viruses. Additionally, conserved cysteine and proline residues, commonly observed in genogroup I PBVs, were present in all three segment 2 sequences. The protein encoded by the ORF2 of two of the segment 2 sequences generated in this study contain ExxRxNxxxE motif, which is reported to be possessed in the corresponding protein in other known PBVs (Da Costa et al., 2011).

#### 3.3.4 Hepesvirus

Hepatitis E virus (HEV) is a non-enveloped, single-stranded RNA virus with a 7.2 kb genome. This study identified a Swine HEV strain within genotype 4, showing 90.5% nucleotide identity with specific Chinese strains (Accession nos. JX893460 and JX893458), while an Indian strain had 78.9% identity with a French isolate (Accession no. MF444087). HEV is of significant global concern due to its prevalence in both developed and developing nations.

## 4 Discussion

Next-generation sequencing (NGS) has revolutionized pathogen discovery through its high throughput, cost-effectiveness, and rapid turnaround. These advances have enabled whole viral genome reconstruction, identification of novel and uncharacterized pathogens, detection of mixed infections, and detailed molecular epidemiology. In clinical microbiology, where conventional diagnostic methods frequently fail to detect complicated or unusual infections, these developments are extremely beneficial (Mardis, 2008; Quick et al., 2016). Though there are still difficulties, especially with clinical and environmental samples, where host nucleic acids frequently make up more than 99% of the composition and obscure microbial sequences. Because of this, it is technically challenging to isolate and identify viral genomes. In this case, metagenomic techniques are crucial, particularly for detecting new viruses and mixed infections. To filter, compile, and analyze large datasets, these methods mostly rely on sophisticated bioinformatics tools.

Viral evolution and horizontal dissemination are facilitated in high-density pig farming systems by factors like overcrowding, the introduction of immunologically naïve piglets, and frequent animal movement. These dynamics increase the probability of zoonotic transmission in addition to having an effect on animal health. Therefore, it is essential to monitor enteric viral diversity, especially in nations like India where there is a scarcity of such data.

In piglets, neonatal diarrhea is a prominent cause of morbidity and mortality, often leading to dehydration and death. Despite being linked to metabolic, viral, or bacterial origins, infectious agents are commonly identified. Co-infections are also frequent, and thorough research on the swine intestine virome is essential for efficient treatment and illness prevention.

Recent metagenomic analyses of the intestinal virome have uncovered numerous novel viral species, many of which remain unstudied, or whose associations with diarrhea are still unclear (Bolger et al., 2014; Maan et al., 2019; Gryaznova et al., 2023; Qian et al., 2024). Our study used a metagenomic NGS approach to profile the fecal virome of diarrheic pigs from a high-density farm in Haryana, India. The sequencing yielded a substantial dataset, revealing high viral diversity. A large proportion of reads were attributed to *Picobirnavirus*, followed by *Sapelovirus* A, *Enterovirus* G, *Aichivirus* C (Porcine Kobuvirus-1), and several unclassified viruses. Importantly, 19.51% of sequences showed no significant match to known GenBank entries, suggesting the presence of novel or poorly characterized viruses. The findings show the co-existence of multiple enteric viruses in the same host, raising concerns about recombination, and reassortment events (Bolger et al., 2014; Theuns et al., 2018; Chen et al., 2022).

Other important detections included Porcine kobuvirus and posavirus, both genetically diverse and with emerging significance (Mohanty et al., 2023). Porcine kobuvirus sequence obtained in this study showed close relation to the kobuvirus strain from Japan. The phylogenetic analysis of porcine kobuvirus revealed its association with both healthy and diarrheic pigs indicating the need for thorough molecular characterization (Jackova et al., 2017). A partial genome of posavirus retrieved in this study, showed a high nucleotide identity with American and Chinese isolates.

Furthermore, the ongoing reclassification of porcine enteric picornaviruses into distinct genera: porcine teschovirus, porcine sapelovirus, and porcine enterovirus G highlights the dynamic nature of viral taxonomy (Zell et al., 2017; Patel et al., 2022). Historically, these enteric viruses have been reported globally, affecting both domestic and wild pigs and leading to a range of diseases from asymptomatic cases to severe syndromes. A previous study revealed that enteric picornaviruses exhibit varying virulence, contributing to numerous pig health issues in India, although direct associations with porcine enteric picornaviruses remain poorly understood (Sawant et al., 2020).

Hepatitis E virus (HEV) genotype 4 was also detected, a genotype known for its zoonotic potential. Given the growing recognition of swine as reservoirs for HEV, this finding is especially important for public health surveillance and farm biosecurity to reduce the risk of interspecies transmission. The potential for co-infections and viral reassortment further complicates the public health landscape, as these dynamics may lead to the emergence of new viral strains with zoonotic capabilities.

Low-titer detection of rotaviruses, picobirnaviruses and porcine parvovirus-7 (PPV-7), also highlights the need for broader diagnostics to capture the full virome. PPV-7 belonging to the *Chaphamaparvovirus* genus and the newly established *Hamaparvovirinae* subfamily and has not been previously reported in India. The detection of PPV-7 in various populations highlights the need for further exploration of its epidemiology and potential impact on porcine health. Our results revealed short contigs of different genes of porcine rotavirus A, and porcine rotavirus C, underscoring the interconnectedness of rotavirus strains across geographical boundaries. Although zoonotic spillover of porcine RVA strains to humans is sporadic, but it has been detected worldwide (Kunić et al., 2023).

Porcine astroviruses (PoAstVs) have garnered considerable attention due to their established association with gastrointestinal diseases in pigs. Within the genus *Mamastrovirus*, five distinct genotypes of PoAstV have been identified (Boujon et al., 2017). The porcine astrovirus identified in our study is closely related to porcine mamastrovirus 3 isolate from China. Recent metagenomic sequencing efforts have revealed the presence of PoAstVs in a range of countries (Blomström et al., 2014; Zhang et al., 2014; Karlsson et al., 2016; Padmanabhan and Hause, 2016; Theuns et al., 2016; Chen et al., 2018). A phylogenetic analysis involving diarrheic piglets in China from 2015 to 2018 identified 27 distinct PAstV strains belonging to three different genotypes (Su et al., 2020).

The increasing use of metagenomic analysis techniques has facilitated the identification of various circular viruses in fecal samples taken from pigs with diarrhea. Such studies have identified porcine circovirus 2 (PCV2) as a significant pathogen causing considerable economic losses in the global pig industry, associated with Porcine Circovirus Disease. The detection and emergence of porcine circovirus 3 (PCV3) in this study represents a critical concern due to its proposed pathogenicity and the lack of cross-protection with PCV2, emphasizing the urgent need for novel vaccines against PCV3 (Pranoto et al., 2023). In this study, the PCV found to exhibit complete nt identity (100%) with Chinese isolates of porcine circovirus 3, while some strains, such as HeN-7C, showed a slightly lower nt identity of 99.6%. The molecular positivity rate of PCV3 in southern India stood at 0.7%, with positive samples linked to reproductive failures for the first time in the region (Bera et al., 2020). Previous studies have indicated that PCV3 is associated with reproductive failure in sows and mortality in piglets, suggesting its significance as a circulating pathogen in apparently healthy pig populations (Bera et al., 2020).

Porcine astrovirus (Mamastrovirus 3) detected in this study has been implicated not only in gastrointestinal symptoms but also in respiratory illness, neurological signs in piglets and even in healthy pigs. This virus, along with the detection of PPV-7 and PCV-3, emphasizes the complex viral ecosystem in swine. These results are consistent with earlier research that used platforms like MinION and found that viruses including *Kobuvirus*, *Enterovirus*, and *Astrovirus* were found in diarrheal pigs, frequently as co-infections (Theuns et al., 2018).

The *Picobirnaviridae* family, which includes porcine picobirnaviruses (PBVs), is characterized by considerable genetic diversity and a broad host range (Knox et al., 2018). PBVs are currently classified into two genogroups based upon classification of a 200 nt sequence of RdRp. But this phylogenetic marker is now saturated, affected by homoplasy, and has high phylogenetic noise, resulting in 34% unsolved topologies (Perez et al., 2023). By contrast, full-length RdRp sequences and deduced amino acid sequences provide reliable topologies that allow ancestralism of members to be correctly inferred. Indeed, several studies have commented on the extremely high degree of sequence and amino acid incongruence among sequences identified as picobirnavirus, which may be as low as 49% similar (Knox et al., 2018). Hence, the use of amino acid sequence alignments and phylogenetic analyses, and predicted protein secondary folding structure model comparisons to determine viral relationships provide some support for the use of a short region that are conserved and taxonomically informative in broad taxonomic classification.

Our phylogenetic analysis confirmed the existence of high genetic diversity within PBVs, with RdRp sequences showing nucleotide identities ranging from 31.3% to 95.1% compared to other genogroup I PBV strains. The conservation of a key motif (GDD) in RdRp domains emphasizes the functional significance of these genes across different PBV strains (Malik et al., 2014a). The presence of multiple PBV segments raises concerns about reassortment and evolutionary dynamics, necessitating closer examination of their potential for interspecies transmission. This is underscored by the observed genetic similarities between animal PBVs and human strains identified in sewage, indicating zoonotic risks.

In summary, our research offers a comprehensive overview of the fecal virome in Indian diarrheic pigs. Out of several identified viruses here, five viruses namely PCV, PEV-G, PSV-A, PKoV and HEV genotype 4 have been previously reported in India. Conversely, the PPV-7, Porcine mamastrovirus 3, and porcine Posavirus, represent novel findings for the Indian context. Despite limitations like small sample size, lack of healthy controls, and geographic restrictions, this study highlights the presence of variety of enteric viruses and their possible effects on pig health, food safety and zoonotic risk. Several published evidence shows that some of the viruses including PCV-2, PCV-3, PPV-7, PSV-A, and PEV-G have been detected in healthy, asymptomatic pigs, indicating that their presence is not exclusively tied to disease (Ni et al., 2017; Zhai et al., 2019; Boros et al., 2021). However, there is no documented evidence of circulation of these viruses in humans. While the absence of healthy controls in our study precludes direct comparative analysis, our findings add to the growing body of evidence describing the complex viral ecology in the porcine gut, particularly under diarrhoeic conditions. We believe that the high abundance and complete genome recovery of viruses such as Porcine Sapelovirus A and Mamastrovirus 3 from these diarrheic samples may indicate active viral replication, and raises important questions about their potential involvement in gastrointestinal disease. But in the absence of a control group comprising healthy piglets, no definitive conclusions can be drawn about the pathogenic role of the detected viruses. In order to improve our knowledge of viral ecology in pigs under the One Health concept, future research should incorporate bigger sample sizes, healthy control animals, and broader geographic coverage. These findings are expected to inform further studies for public health policy and farming practices. This includes advocating for comprehensive surveillance programs to monitor viral infections in both swine and humans, which can inform broader public health initiatives aimed at prevention and control.

## 5 Conclusion

This study offers valuable insights into the enteric virome of pigs with diarrhea in India and demonstrates the utility of metagenomic sequencing in detecting both known and novel RNA and DNA viruses. The identification of multiple viral pathogens including those with zoonotic potential highlights the need for continued surveillance, particularly with broader sampling, inclusion of healthy controls, and deeper genomic characterization to inform both veterinary health and public health strategies.

## Data Availability

Sequence reads were deposited in GenBank under the BioSample accession number: SAMN37033531.

## References

[B1] Altschul S. F. Madden T. L. Schäffer A. A. Zhang J. Zhang Z. Miller W.. (1997). Gapped BLAST and PSI-BLAST: A new generation of protein database search programs. Nucleic Acids Res. 25, 3389–3402. doi: 10.1093/nar/25.17.3389, PMID: 9254694 PMC146917

[B2] Andrews S. (2010). FastQC: A quality control tool for high throughput sequence data. Available online at: https://www.bioinformatics.babraham.ac.uk/projects/fastqc/ (Accessed February 28, 2025).

[B3] Bányai K. Potgieter C. Gellért Á. Ganesh B. Tempesta M. Lorusso E.. (2014). Genome sequencing identifies genetic and antigenic divergence of porcine picobirnaviruses. J. Gen. Virol. 95, 2233–2239. doi: 10.1099/vir.0.057984-0, PMID: 24584476

[B4] Bera B. C. Choudhary M. Anand T. Virmani N. Sundaram K. Choudhary B.. (2020). Detection and genetic characterization of porcine circovirus 3 (PCV3) in pigs in India. Transbound Emerg. Dis. 67, 1062–1067. doi: 10.1111/tbed.13463, PMID: 31880100

[B5] Bhattacharya R. Sahoo G. C. Nayak M. K. Rajendran K. Dutta P. Mitra U.. (2007). Detection of Genogroup I and II human picobirnaviruses showing small genomic RNA profile causing acute watery diarrhoea among children in Kolkata, India. Infect. Genet. Evol. 7, 229–238. doi: 10.1016/j.meegid.2006.09.005, PMID: 17049316

[B6] Blomström A. L. Ley C. Jacobson M. (2014). Astrovirus as a possible cause of congenital tremor type AII in piglets? Acta Vet. Scand. 56(1), 82. doi: 10.1186/s13028-014-0082-y, PMID: 25510194 PMC4271328

[B7] Bodewes R. Rubio García A. Wiersma L. C. Getu S. Beukers M. Schapendonk C. M.. (2013). Novel B19-like parvovirus in the brain of a harbor seal. PloS One 8, e79259. doi: 10.1371/journal.pone.0079259, PMID: 24223918 PMC3818428

[B8] Bolger A. M. Lohse M. Usadel B. (2014). Trimmomatic: A flexible read trimming tool for Illumina NGS data. Bioinformatics 30, 2114–2120. doi: 10.1093/bioinformatics/btu170, PMID: 24695404 PMC4103590

[B9] Boros Á. Marton S. Palya V. Egyed L. (2021). Prevalence of porcine sapelovirus A in healthy and diarrhoeic pigs and wild boars in Central Europe. Arch. Virol. 166, 2761–2767. doi: 10.1007/s00705-021-05145-z

[B10] Boujon C. L. Selimovic-Hamza S. Bouzalas I. Seuberlich T. (2017). Development and validation of an immunohistochemistry procedure for the detection of a neurotropic bovine astrovirus. J. Virol. Methods 239, 26–33. doi: 10.1016/j.jviromet.2016.10.013, PMID: 27916667

[B11] Chelli E. De Sabato L. Vaccari G. Ostanello F. Di Bartolo I. (2020). Detection and characterization of porcine sapelovirus in Italian pig farms. Animals 10, 966. doi: 10.3390/ani10060966, PMID: 32498384 PMC7341194

[B12] Chen Y. M. Sadiq S. Tian J. H. Chen X. Lin X. D. Shen J. J.. (2022). RNA viromes from terrestrial sites across China expand environmental viral diversity. Nat. Microbiol. 7, 1312–1323. doi: 10.1038/s41564-022-01180-2, PMID: 35902778

[B13] Chen M. Sun H. Lan D. Hua X. Cui L. Yuan C.. (2014). Molecular detection of Genogroup I and II picobirnaviruses in pigs in China. Virus Genes 48, 553–556. doi: 10.1007/s11262-014-1058-8, PMID: 24682937

[B14] Chen Q. Wang L. Zheng Y. Zhang J. Guo B. Yoon K. J.. (2018). Metagenomic analysis of the RNA fraction of the fecal virome indicates high diversity in pigs infected by porcine endemic diarrhea virus in the United States. Virol. J. 15, 95. doi: 10.1186/s12985-018-1001-z, PMID: 29801460 PMC5970503

[B15] Chiu C. Y. Miller S. A. (2019). Clinical metagenomics. Nat. Rev. Genet. 20, 341–355. doi: 10.1038/s41576-019-0113-7, PMID: 30918369 PMC6858796

[B16] Cortey M. Díaz I. Vidal A. Martín-Valls G. Franzo G. Gómez de Nova P. J.. (2019). High levels of unreported intraspecific diversity among RNA viruses in faeces of neonatal piglets with diarrhoea. BMC Vet. Res. 15, 441. doi: 10.1186/s12917-019-2204-2, PMID: 31805938 PMC6896758

[B17] Da Costa B. Duquerroy S. Tarus B. Delmas B. (2011). Picobirnaviruses encode a protein with repeats of the ExxRxNxxxE motif. Virus Res. 158, 251–256. doi: 10.1016/j.virusres.2011.02.018, PMID: 21376090

[B18] Delmas B. Attoui H. Ghosh S. Malik Y. S. Mundt E. Vakharia V. N. ICTV Report Consortium. (2019). ICTV virus taxonomy profile: Picobirnaviridae. J. Gen. Virol. 100, 133–134. doi: 10.1099/jgv.0.001186, PMID: 30484763 PMC12662030

[B19] Edgar R. C. (2004). MUSCLE: multiple sequence alignment with high accuracy and high throughput. Nucleic Acids Res. 32, 1792–1797. doi: 10.1093/nar/gkh340, PMID: 15034147 PMC390337

[B20] Ganesh B. Bányai K. Martella V. Jakab F. Masachessi G. Kobayashi N. (2012). Picobirnavirus infections: viral persistence and zoonotic potential. Rev. Med. Virol. 22, 245–256. doi: 10.1002/rmv.1707, PMID: 22311513

[B21] Ganesh B. Nagashima S. Ghosh S. Nataraju S. M. Rajendran K. Manna B.. (2011). Detection and molecular characterization of multiple strains of Picobirnavirus causing mixed infection in a diarrhoeic child: emergence of prototype Genogroup II-like strain in Kolkata, India. Int. J. Mol. Epidemiol. Genet. 2, 61–72., PMID: 21537403 PMC3077240

[B22] Gillman L. Sánchez A. M. Arbiza J. (2013). Picobirnavirus in captive animals from Uruguay: identification of new hosts. Intervirology 56, 46–49. doi: 10.1159/000338275, PMID: 22759924

[B23] Giordano M. O. Martinez L. C. Rinaldi D. Gúinard S. Naretto E. Casero R.. (1998). Detection of picobirnavirus in HIV-infected patients with diarrhea in Argentina. J. Acquir. Immune Defic. Syndr. Hum. Retrovirol. 18, 380–383. doi: 10.1097/00042560-199808010-00010, PMID: 9704944

[B24] Grant J. R. Enns E. Marinier E. Mandal A. Herman E. K. Chen C.-Y.. (2023). Proksee: In-depth characterization and visualization of bacterial genomes. Nucleic Acids Res. 51, W484–W492. doi: 10.1093/nar/gkad326, PMID: 37140037 PMC10320063

[B25] Gryaznova M. Smirnova Y. Burakova I. Morozova P. Nesterova E. Gladkikh M.. (2023). Characteristics of the fecal microbiome of piglets with diarrhea identified using shotgun metagenomics sequencing. Animals 13, 2303. doi: 10.3390/ani13142303, PMID: 37508080 PMC10376196

[B26] Hause B. M. Hesse R. A. Anderson G. A. (2015). Identification of a novel Picornavirales virus distantly related to posavirus in swine feces. Virus Genes 51, 144–147. doi: 10.1007/s11262-015-1215-8, PMID: 26032164

[B27] Hause B. M. Palinski R. Hesse R. Anderson G. (2016). Highly diverse posaviruses in swine faeces are aquatic in origin. J. Gen. Virol. 97, 1362–1367. doi: 10.1099/jgv.0.000461, PMID: 27002315

[B28] Höper D. Wylezich C. Beer M. (2017). Loeffler 4.0: diagnostic metagenomics. Adv. Virus Res. 99, 17–37. doi: 10.1016/bs.aivir.2017.08.001, PMID: 29029726 PMC7112322

[B29] Jackova A. Sliz I. Mandelik R. Salamunova S. Novotny J. Kolesarova M.. (2017). Porcine kobuvirus 1 in healthy and diarrheic pigs: Genetic detection and characterization of virus and co-infection with rotavirus A. Infect. Genet. Evol. 49, 73–77. doi: 10.1016/j.meegid.2017.01.011, PMID: 28087494

[B30] Karlsson O. E. Larsson J. Hayer J. Berg M. Jacobson M. (2016). The intestinal eukaryotic virome in healthy and diarrhoeic neonatal piglets. PloS One 11, e0151481. doi: 10.1371/journal.pone.0151481, PMID: 26982708 PMC4794121

[B31] Knox M. A. Gedye K. R. Hayman D. T. S. (2018). The challenges of analysing highly diverse picobirnavirus sequence data. Viruses 10, 685. doi: 10.3390/v10120685, PMID: 30513931 PMC6316005

[B32] Kumar S. Stecher G. Tamura K. (2016). MEGA7: Molecular evolutionary genetics analysis version 7.0 for bigger datasets. Mol. Biol. Evol. 33, 1870–1874. doi: 10.1093/molbev/msw054, PMID: 27004904 PMC8210823

[B33] Kunić V. Mikuletič T. Kogoj R. Koritnik T. Steyer A. Šoprek S.. (2023). Interspecies transmission of porcine-originated G4P[6] rotavirus A between pigs and humans: a synchronized spatiotemporal approach. Front. Microbiol. 14. doi: 10.3389/fmicb.2023.1194764, PMID: 37283926 PMC10239803

[B34] Langmead B. Salzberg S. L. (2012). Fast gapped-read alignment with Bowtie 2. Nat. Methods 9, 357–359. doi: 10.1038/nmeth.1923, PMID: 22388286 PMC3322381

[B35] Letunic I. Bork P. (2021). Interactive Tree Of Life (iTOL) v5: an online tool for phylogenetic tree display and annotation. Nucleic Acids Res. 49, W293–W296. doi: 10.1093/nar/gkab301, PMID: 33885785 PMC8265157

[B36] Maan S. Chaudhary D. Bansal N. Devi B. Maan N. S. (2019). Development of next generation sequencing protocols for dsRNA viruses. Int. J. Curr. Microbiol. Appl. Sci. 8, 1062–1067. doi: 10.20546/ijcmas.2019.802.125

[B37] Malik Y. S. Kumar N. Sharma K. Dhama K. Shabbir M. Z. Ganesh B.. (2014a). Epidemiology, phylogeny, and evolution of emerging enteric picobirnaviruses of animal origin and their relationship to human strains. Biomed. Res. Int. 2014, 780752. doi: 10.1155/2014/780752, PMID: 25136620 PMC4124650

[B38] Malik Y. S. Sharma A. K. Kumar N. Sharma K. Ganesh B. Kobayashi N. (2014b). Identification and characterisation of a novel Genogroup II picobirnavirus in a calf in India. Vet. Rec. 174, 278. doi: 10.1136/vr.102065, PMID: 24570405

[B39] Mardis E. R. (2008). Next-generation DNA sequencing methods. Annu. Rev. Genomics Hum. Genet. 9, 387–402. doi: 10.1146/annurev.genom.9.081307.164359, PMID: 18576944

[B40] Masachessi G. Ganesh B. Martinez L. C. Giordano M. O. Barril P. A. Isa M. B.. (2015). Maintenance of picobirnavirus (PBV) infection in an adult orangutan (Pongo pygmaeus) and genetic diversity of excreted viral strains during a three-year period. Infect. Genet. Evol. 29, 196–202. doi: 10.1016/j.meegid.2014.11.019, PMID: 25435283

[B41] Midgley S. E. Bányai K. Buesa J. Halaihel N. Hjulsager C. K. Jakab F.. (2012). Diversity and zoonotic potential of rotaviruses in swine and cattle across Europe. Vet. Microbiol. 156(3-4), 238–245. doi: 10.1016/j.vetmic.2011.10.027, PMID: 22079216

[B42] Mohanty P. Panda P. Acharya R. K. Pande B. Bhaskar L. Verma H. K. (2023). Emerging perspectives on RNA virus-mediated infections: from pathogenesis to therapeutic interventions. World J. Virol. 12, 242–255. doi: 10.5501/wjv.v12.i5.242, PMID: 38187500 PMC10768389

[B43] Mondal A. Chakravarti S. Majee S. B. Bannalikar A. S. (2013). Detection of picobirnavirus and rotavirus in diarrhoeicfaecal samples of cattle and buffalo calves in Mumbai metropolis, Western India. Vet. Ital. 49, 357–360. doi: 10.12834/VetIt.1109.10, PMID: 24362776

[B44] Ni Y. Wang Z. Ye X. Yang F. Xu J. Hung T.. (2017). Sequence and phylogenetic analysis of a novel porcine parvovirus 7 strain detected in fecal samples from healthy adult pigs in the USA. Arch. Virol. 162, 2345–2350. doi: 10.1007/s00705-017-3358-z 28462462

[B45] Ondov B. D. Bergman N. H. Phillippy A. M. (2011). Interactive metagenomic visualization in a web browser. BMC Bioinf. 12, 385. doi: 10.1186/1471-2105-12-385, PMID: 21961884 PMC3190407

[B46] Oude Munnink B. B. Cotten M. Deijs M. Jebbink M. F. Bakker M. JazaeriFarsani S. M.. (2015). A novel genus in the order Picornavirales detected in human stool. J. Gen. Virol. 96, 3440–3443. doi: 10.1099/jgv.0.000279, PMID: 26354795

[B47] Oude Munnink B. B. Phan M. V. T. Consortium VIZIONS Simmonds P. Koopmans M. P. G. Kellam P.. (2017). Characterization of Posa and Posa-like virus genomes in fecal samples from humans, pigs, rats, and bats collected from a single location in Vietnam. Virus Evol. 3, vex022. doi: 10.1093/ve/vex022, PMID: 28948041 PMC5597861

[B48] Padmanabhan A. Hause B. M. (2016). Detection and characterization of a novel genotype of porcine astrovirus 4 from nasal swabs from pigs with acute respiratory disease. Arch. Virol. 161, 2575–2579. doi: 10.1007/s00705-016-2937-1, PMID: 27329081

[B49] Patel S. K. Agrawal A. Pathak M. Singh A. Varshney R. Rana J.. (2022). Detection of porcine enteric picornaviruses from faecal samples of Indian pigs. Virus Dis. 33, 102–107. doi: 10.1007/s13337-022-00756-0, PMID: 35493750 PMC9005585

[B50] Perez L. J. Cloherty G. A. Berg M. G. (2023). Parallel evolution of picobirnaviruses from distinct ancestral origins. Microbiol. Spectr. 11, e0269323. doi: 10.1128/spectrum.02693-23, PMID: 37888988 PMC10714727

[B51] Pranoto S. Wu H. C. Chun-Yen C. (2023). Porcine circovirus type 3: Diagnostics, genotyping, and challenges in vaccine development. Transbound Emerg. Dis. 858447, 9. doi: 10.1155/2023/8858447, PMID: 40303716 PMC12016748

[B52] Prjibelski A. Antipov D. Meleshko D. Lapidus A. Korobeynikov A. (2020). Using SPAdes *de novo* assembler. In: Shen, M. (Ed.). Gene Predict 2067, 233–254. doi: 10.1007/978-1-4939-9940-8_9, PMID: 32559359

[B53] Qian L. Zhuang Z. Lu J. Wang H. Wang X. Yang S.. (2024). Metagenomic survey of viral diversity obtained from feces of piglets with diarrhea. Heliyon 10, e25616. doi: 10.1016/j.heliyon.2024.e25616, PMID: 38375275 PMC10875384

[B54] Quick J. Loman N. J. Duraffour S. Simpson J. T. Severi E. Cowley L.. (2016). Real-time, portable genome sequencing for Ebola surveillance. Nature 530, 228–232. doi: 10.1038/nature16996, PMID: 26840485 PMC4817224

[B55] Reuter J. A. Spacek D. V. Snyder M. P. (2015). High-throughput sequencing technologies. Mol. Cell 58, 586–597. doi: 10.1016/j.molcel.2015.05.004, PMID: 26000844 PMC4494749

[B56] Rosen B. I. Fang Z. Y. Glass R. I. Monroe S. S. (2000). Cloning of human picobirnavirus genomic segments and development of an RT-PCR detection assay. Virology 277, 316–329. doi: 10.1006/viro.2000.0594, PMID: 11080479

[B57] Sachsenroder J. Twardziok S. O. Scheuch M. Johne R. (2014). The general composition of the faecalvirome of pigs depends on age, but not on feeding with a probiotic bacterium. PloS One 9, e88888. doi: 10.1371/journal.pone.0088888, PMID: 24586429 PMC3929612

[B58] Sawant P. M. Atre N. Kulkarni A. Gopalkrishna V. (2020). Detection and molecular characterization of porcine enterovirus G15 and teschovirus from India. Pathog. Dis. 78, ftaa039. doi: 10.1093/femspd/ftaa039, PMID: 32691821

[B59] Sawant P. Kulkarni A. Mane R. Patil R. Lavania M. (2023). Metatranscriptomic assessment of diarrhoeicfaeces reveals diverse RNA viruses in rotavirus group A infected piglets and calves from India. Front. Cell. Infect. Microbiol. 13. doi: 10.3389/fcimb.2023.1258660, PMID: 37965252 PMC10642067

[B60] Shan T. Li L. Simmonds P. Wang C. Moeser A. Delwart E. (2011). The fecal virome of pigs on a high-density farm. J. Virol. 85, 11697–11708. doi: 10.1128/JVI.05217-11, PMID: 21900163 PMC3209269

[B61] Smits S. L. Schapendonk C. M. van Beek J. Vennema H. Schürch A. C. Schipper D.. (2014). New viruses in idiopathic human diarrhea cases, the Netherlands. Emerg. Infect. Dis. 20, 1218–1222. doi: 10.3201/eid2007.140190, PMID: 24964003 PMC4073879

[B62] Smoľak D. Šalamúnová S. Jacková A. Haršányová M. Budiš J. Szemes T.. (2022). Analysis of RNA virome in rectal swabs of healthy and diarrheic pigs of different age. Comp. Immunol. Microbiol. Infect. Dis. 90–91:101892. doi: 10.1016/j.cimid.2022.101892, PMID: 36274336

[B63] Su M. Qi S. Yang D. Guo D. Yin B. Sun D. (2020). Coinfection and genetic characterization of porcine astrovirus in diarrheic piglets in China from 2015 to 2018. Front. Vet. Sci. 7. doi: 10.3389/fvets.2020.00462, PMID: 32923463 PMC7456941

[B64] Theuns S. Conceição-Neto N. Zeller M. Heylen E. Roukaerts I. D. Desmarets L. M.. (2016). Characterization of a genetically heterogeneous porcine rotavirus C, and other viruses present in the fecal virome of a non-diarrheic Belgian piglet. Infect. Genet. Evol. 43, 135–145. doi: 10.1016/j.meegid.2016.05.018, PMID: 27184192 PMC7172746

[B65] Theuns S. Vanmechelen B. Bernaert Q. Deboutte W. Vandenhole M. Beller L.. (2018). Nanopore sequencing as a revolutionary diagnostic tool for porcine viral enteric disease complexes identifies porcine kobuvirus as an important enteric virus. Sci. Rep. 8, 9830. doi: 10.1038/s41598-018-28180-9, PMID: 29959349 PMC6026206

[B66] Thurber R. V. Haynes M. Breitbart M. Wegley L. Rohwer F. (2009). Laboratory procedures to generate viral metagenomes. Nat. Protoc. 4, 470–483. doi: 10.1038/nprot.2009.10, PMID: 19300441

[B67] Verma H. Mor S. K. Erber J. Goyal S. M. (2015). Prevalence and complete genome characterization of Turkey picobirnaviruses. Infect. Genet. Evol. 30, 134–139. doi: 10.1016/j.meegid.2014.12.014, PMID: 25530436 PMC7172272

[B68] Wakuda M. Pongsuwanna Y. Taniguchi K. (2005). Complete nucleotide sequences of two RNA segments of human picobirnavirus. J. Virol. Methods 126, 165–169. doi: 10.1016/j.jviromet.2005.02.010, PMID: 15847933

[B69] Woo P. C. Y. Teng J. L. L. Bai R. Tang Y. Wong A. Y. P. Li K. S. M.. (2019). Novel picobirnaviruses in respiratory and alimentary tracts of cattle and monkeys with large intra- and inter-host diversity. Viruses 11, 574. doi: 10.3390/v11060574, PMID: 31234565 PMC6631280

[B70] Woo P. C. Y. Teng J. L. L. Bai R. Wong A. Y. P. Martelli P. Hui S. W.. (2016 1886). High diversity of Genogroup I picobirnaviruses in mammals. Front. Microbiol. 7. doi: 10.3389/fmicb.2016.01886, PMID: 27933049 PMC5120130

[B71] Wood D. E. Salzberg S. L. (2014). Kraken: Ultrafast metagenomic sequence classification using exact alignments. Genome Biol. V15, R46. doi: 10.1186/gb-2014-15-3-r46, PMID: 24580807 PMC4053813

[B72] Zell R. Delwart E. Gorbalenya A. E. Hovi T. King A. M. Q. Knowles N. J.. (2017). ICTV virus taxonomy profile: picornaviridae. J. Gen. Virol. 98, 2421–2422. doi: 10.1099/jgv.0.000911, PMID: 28884666 PMC5725991

[B73] Zhai S. L. Cheung A. K. Zhang H. B. Long J. X. Yuan S. S. (2019). Prevalence and molecular evolution of PCV3 in pigs: viral persistence and infection in asymptomatic animals. Sci. Rep. 9, 10241. doi: 10.1038/s41598-019-46640-0 31308406

[B74] Zhang B. Tang C. Yue H. Ren Y. Song Z. (2014). Viral metagenomics analysis demonstrates the diversity of viral flora in piglet diarrhoeic faeces in China. J. Gen. Virol. 95, 1603–1611. doi: 10.1099/vir.0.063743-0, PMID: 24718833

[B75] Zhang Z. Wang Y. Wang J. Wang J. Pan Y. (2019). VGAS: A viral genome annotation system. Front. Microbiol. 10. doi: 10.3389/fmicb.2019.00184, PMID: 30814982 PMC6381048

