# Supplementary material for: Exploring viral diversity in diarrheic porcine feces: a metagenomic analysis from an Indian swine farm
Source: Front Cell Infect Microbiol. 2025 Sep 12;15:1653342. doi: 10.3389/fcimb.2025.1653342 (PMC12463912; doi:10.3389/fcimb.2025.1653342)
Supplement: Supplementary file 1 [file Table1.doc]

**Supplementary Table 1. Closest homologs and their hosts of the identified viral genome sequences.**

| **Viral genome** | **Closest homolog** | **% of Identity** | **Host species** |
| --- | --- | --- | --- |
| *Porcine Sapelovirus A* | *Porcine sapelovirus 1* isolate IVRI/PSV/SPF | 88.4% | *Sus scrofa* |
| *Porcine enterovirus G* | *Porcine enterovirus G* isolate CH/GXQZ/2017 | 100% | *Sus scrofa domesticus* |
| *Porcine kobuvirus* | *Porcine kobuvirus* gene for polyprotein,strain: PoKoV/Iba444-2/JPN/2016 | 90.6% | *Sus scrofa* |
| *Posavirus* | *Posavirus 1* strain 9470 polyprotein gene, partial cds | 100% | *Sus scrofa domesticus* |
| *Porcine mamastrovirus* 3 | Mamastrovirus 3 isolate PAstV-GX1 | 86.3% | *Sus scrofa domesticus* |
| *Porcine circovirus* 3 | *Porcine circovirus* isolate PCV3/Pig/CN/ShanXi170709 | 100% | *Sus scrofa domesticus* |
|  | *Porcine circovirus* 3 isolate FJ37 | 100% | *Sus scrofa domesticus* |
|  | *Porcine circoviru*s 3 strain PCV3-China/JL16-38 | 100% | *Sus scrofa domesticus* |
| *Porcine parvovirus* 7 (701 nt) | *Porcine parvovirus* 7 isolate GX14-1998 | 96.3% | *Sus scrofa domesticus* |
|  | *Porcine parvovirus* 7 strain 21FJ13 | 96.3% | *Sus scrofa domesticus* |
| *Porcine parvovirus* 7 (857 nt) | *Porcine parvovirus* 7 strain 20FJSM34 | 96.1% | *Sus scrofa domesticus* |
| *Porcine rotavirus* (RVA) | *Porcine rotavirus* isolate RVA/Pig/China/FJSH01/2021/G26P[23] | 96.4% | *Sus scrofa* |
| *Porcine rotavirus* (RVC) | *Porcine rotavirus* strain F7P4 VP1 gene | 92.3% | *Sus scrofa* |
| *Porcine picobirnavirus* (Capsid Protein) | Capsid, partial [*Macaque picobirnavirus* 6] | 87.87% | *Macaca mulatta* |
| *Porcine picobirnavirus* (RdRp Protein) | MAG: putative RNA-dependent RNA polymerase, partial [*Picobirnaviridae* sp.] | 99.65% | *Sus scrofa domesticus* |
| *Hepatitis E virus* | Swine *hepatitis E virus* isolate swCNAH2581-10 replicase gene, partial cds. | 90.5% | *Sus scrofa domesticus* |
